# A novel ZVS full-bridge cascaded step-up DC-DC converter with resonant auxiliary circuit for high voltage-gain applications

**DOI:** 10.1371/journal.pone.0306906

**Published:** 2024-08-15

**Authors:** M. Zakir Hossain, Jeyraj A/L Selvaraj, N. A. Rahim

**Affiliations:** 1 Department of Electrical and Electronic Engineering, Dhaka University of Engineering & Technology, Gazipur, Bangladesh; 2 UM Power Energy Dedicated Advanced Centre (UMPEDAC), HICoE, Wisma R&D, University of Malaya, Kuala Lumpur, Malaysia; Federal University of Technology - Parana, BRAZIL

## Abstract

High conversion ratio dc-dc converters have received significant attention in renewable energy systems, primarily due to their necessary high-gain characteristics. This research proposes a high step-up ratio full-bridge resonant cascaded (FBRC) dc-dc converter designed for use in photovoltaics (PV), fuel cells (FC), electric vehicles (EV), and other low-voltage output energy sectors to achieve high voltage gain. This converter contains a full-bridge cell with a boost input inductor, a diode-capacitor cascaded stage that replaces the transformer as a voltage multiplier and an inductor-capacitor (LC) parallel-series resonant network across the FB terminal. One of the strategic features of the converter is its high voltage step-up characteristic combined with lower duty cycle operation that limits the maximum current through the active devices, making it particularly suitable for systems that generate low output voltage. In addition, zero-voltage switching (ZVS) is achieved during the turn-off and turn-on operation of the FB switches from 25% to full load, thereby lessening the switching losses. Moreover, the diminished necessity for passive components and the decreased voltage stress on both active and passive devices lead to the use of smaller and more cost-effective components. The theoretical analysis of the proposed converter is validated using a 500 W laboratory-scale prototype wherein high-performance SiC-based MOSFETs have been utilized as switching devices. It offers reduced ripples, with input current ripple at 5% and output voltage ripple at 0.76%. When the load is 400 W and 60 V as the input voltage, the maximum efficiency is found 95.8% at 400 V output voltage. The proposed dc-dc converter, with its high voltage gain and reduced component stress, shows significant promise for application in renewable energy systems.

## 1. Introduction

In response to the escalating global energy demand, the use of renewable energy is expanding day by day. Due to its reliability and environmentally friendly nature, governments and policymakers have also prioritized the development of new technologies to address energy needs. Although renewable energy systems like photovoltaic (PV), wind, and fuel cells (FC), etc. have gained significant momentum [1–4], the main obstacle to their growth consists in the low power conversion efficiency. On one hand, the output voltage of both the PV module and fuel cell is very low and the PV power output is directly affected by the diurnal changes in solar intensity. Consequently, a higher-voltage step-up dc-dc converter is necessary to amplify the output and ensure compatibility with other devices or applications [5, 6]. Furthermore, there is a demand for greater voltage-gain dc-dc converters in other applications, in addition to the domains described before. Several instances include offshore wind energy [3], electric, hybrid electric or fuel-cell vehicles [7–9], power systems integrating both medium- and high-voltage DC (MVDC and HVDC) [10, 11], and various other applications relying on fuel cells [12].

High step-up dc-dc converters can be categorized based on their active switches, isolation state, level of efficiency, voltage-gain technique, etc. [13–15]. Each group has some pros and cons. In recent times, a lot of research has been carried out to build non-isolated (transformer-less) topologies to attain high gain [16–18]. These are easier to build with a higher power density (in the absence of a transformer), cost-effective, and simple operation. They are appropriate for applications where electrical isolation is not a primary concern and lower power applications.

Several new research has been performed using high turns ratio transformers to make a high-gain converter for renewable power [19–21]. On the other hand, isolated boost converters with transformers have several merits including the provision of electrical isolation between the input and output, resolving ground loop problems and safety issues, suitable for high-power applications, and improving voltage regulation and less electromagnetic interference (EMI). These characteristics enable their integration into high-power systems with enhanced stability and robust control. Nevertheless, they encounter several challenges, including increased size due to additional isolation components, higher cost, winding losses, elevated leakage inductance, and reduced efficiency as pointed out in [22]. These issues have been tackled by using more active switches for active clamping and some expensive and complicated control methods [23–25].

The PWM dc–dc converters have the most basic isolated designs, which include the Forward, the Flyback, the Push–Pull (PP), and the conventional Ćuk, Sepic, and Zeta with galvanic isolation. However, their static dc voltage step-up ratio isn’t as good as it used to be, and the input current usually has a lot of noise. Also, there is a lot of voltage stress on their output diodes, so they need diodes with a high breakdown voltage rating [26, 27].

Depending on the application, resonant dc-dc converters have advantages and disadvantages in terms of expense, complication, compactness, dependability, and efficiency. Better transformer utilization and strong dc voltage gains are provided by the current-fed resonant converter in [28]. Besides, the transformer’s leakage inductance is integrated into the converter’s basic workings as a resonant network, aiding in zero-current switching (ZCS) for the frond-side inverter switches. With its less component use, this converter is appropriate for low-power systems. In [29], a flyback topology for an RF, ZVS resonant PP converter is presented. It exhibits a high power density despite the high component count. More costs are required to reach a very high power density. This converter offers the same dc voltage gain ratio as the RF ZVS PP quasi-resonant converter in [30]. However, both converters’ dependability declines and complexity rises as a result of their three winding transformers and numerous switches [29, 30].

Many investigations have been conducted to get high gain by the application of the principles of diode-capacitor or diode-inductor cells [31–34]. Their sturdy and straightforward structures make it easy to use simpler control strategies. However, when voltage-cascaded stages increase, the majority of these converters experience increased voltage stress. For instance, the converter in [31], uses a voltage multiplier network based on FB cascaded diode capacitors to achieve a high voltage step-up ratio without the need for high-duty cycle operation. Furthermore, the voltage stress is comparatively smaller and is independent of the change in cascaded stages for capacitors, diodes, and switching devices. Nevertheless, this topology is not widely used because of the hard switching, low efficiency, and large boost inductance. A modified version of the converter [31], is suggested in [35], with an additional small inductance added between the FB legs. It provides cascaded stage independence, lower voltage stress, and a high conversion ratio. Additionally, by using a smaller cascaded capacitance and lower boost inductance, it achieves a slightly greater efficiency than the converter in [31]. However, soft switching operation was not achieved by this architecture.

The literature review indicates a clear and ongoing need for resonant soft-switching high conversion ratio dc-dc converters that can provide substantial voltage gain at lower voltage stress with low ripples, specifically for low-voltage output energy sectors. This article proposes a full-bridge resonant cascaded (FBRC) dc-dc converter that offers minor ripples in output voltage and input current, compactness, improved efficiency, and less voltage stress on the FB switches. Moreover, it provides zero switching losses through the ZVS of the FB switches. Therefore, for low-voltage output sources like fuel cells, PV panels, and other renewable energy systems, this high-voltage step-up ratio converter can be a suitable component. The subsequent sections of this article are structured as follows: Section 2 elucidates the principles governing converter operation. In Section 3, a comparative analysis of performance is provided, coupled with discussions on the design considerations and parameter selection. Section 4 offers a concise depiction of the control mechanism employed in the proposed converter. Section 5 delineates the experimental results and their interpretation. The concluding remarks of the article are presented in Section 6.

## 2. Converter operation

The full-bridge cascaded (FBC) converter reported in [35] has been improved by adding a resonant auxiliary circuit in place of the parallel inductor to achieve higher efficiency and ZVS operation. This section describes how this converter operates based on its different operating modes showing the current flow routes and optimal waveforms for each mode. The following presumptions are taken into account to streamline the analysis and operation smoothly:

1. The converter operates in steady-state mode with *n*-stage of cascaded multiplier and continuous conduction mode (CCM).
2. No losses and ripple are taken into account; all the passive and active devices are ideal.
3. Because every capacitor utilized in this architecture is sufficiently large, every capacitor’s voltage is the same, except the first capacitor’s (*C*_*1*_) value, which is half that of the other capacitors.

**Fig 1:**
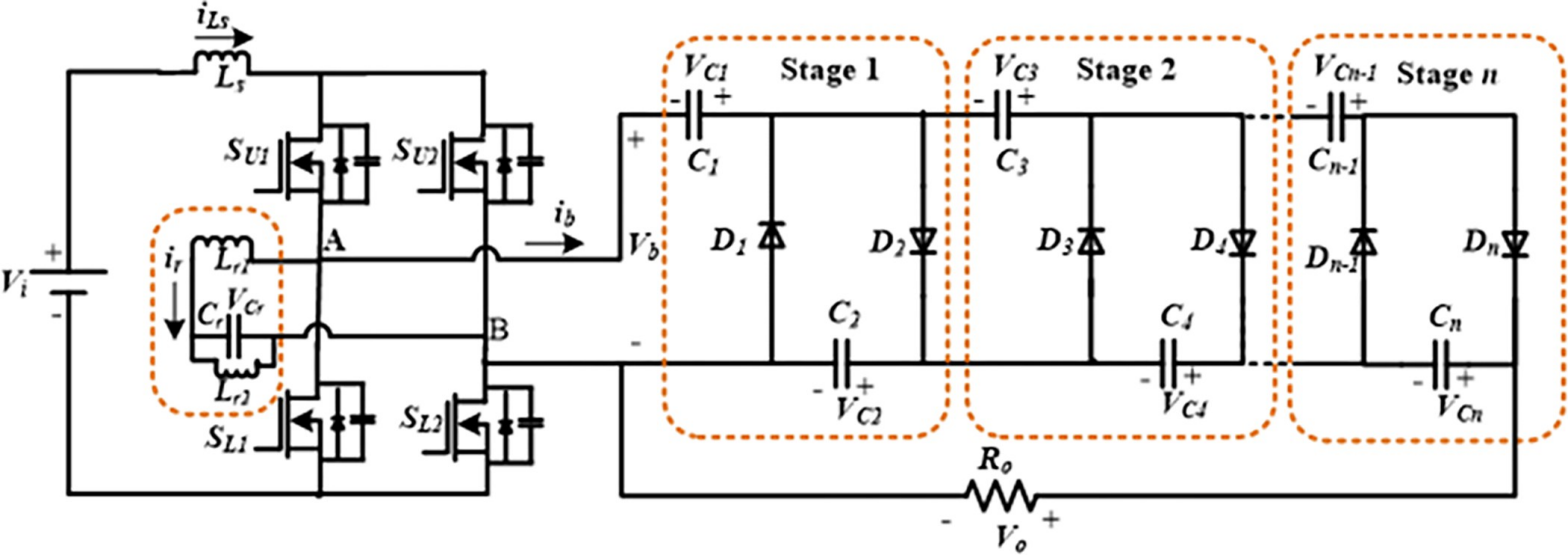
Proposed Full-bridge resonant cascaded (FBRC) step-up dc-dc converter.

Fig 1 shows the developed high step-up FBRC converter. Low-voltage DC sources such as PV modules, FCs, batteries, DC power supplies, etc., can be used as the converter’s input. The converter in Fig 1 is built using an n-stage cascaded voltage multiplier, a resonant branch, and an FB cell comprising four switches designated *S*_*U1*_, *S*_*U2*_, *S*_*L1*_, and *S*_*L2*_, along with a boost input inductor (*L*_*s*_). The resonant inductor (*L*_*r1*_) is connected in series with the parallel capacitor (*C*_*r*_)-inductor (*L*_*r2*_) branch placed between the ac terminals (A and B) of the FB module. A single pair of diodes and a couple of capacitors make up a cascaded stage, whereas an *n*-stage contains 2*n* = *N* diodes and a comparable number of capacitors.

The resonant converter’s switching frequency is maintained close to its resonant frequency for optimal performance. Two distinct, independent frequencies are used to turn on and off the FB switches. Two distinct frequencies, designated as *f*_*SU*_ and *f*_*SL*_, respectively, are used to operate the two upper switches (*S*_*U1*_ and *S*_*U2*_) and the two bottom switches (*S*_*L1*_ and *S*_*L2*_). A higher current can be supplied by the resonant branch to charge the FB switches’ bypass capacitors, achieving ZVS for both the upper and lower switches. To get the desired performance, the *f*_*SU*_ is kept substantially lower than the *f*_*SL*_ in this study, while the *f*_*SL*_ is chosen as close to the resonant frequency as feasible. While *f*_*SU*_ is maintained at a fixed value to achieve the necessary output voltage ripple, the duty cycle of *f*_*SL*_ is adjusted within the resonance region to control the output voltage, *V*_*o*_.

**Fig 2:**
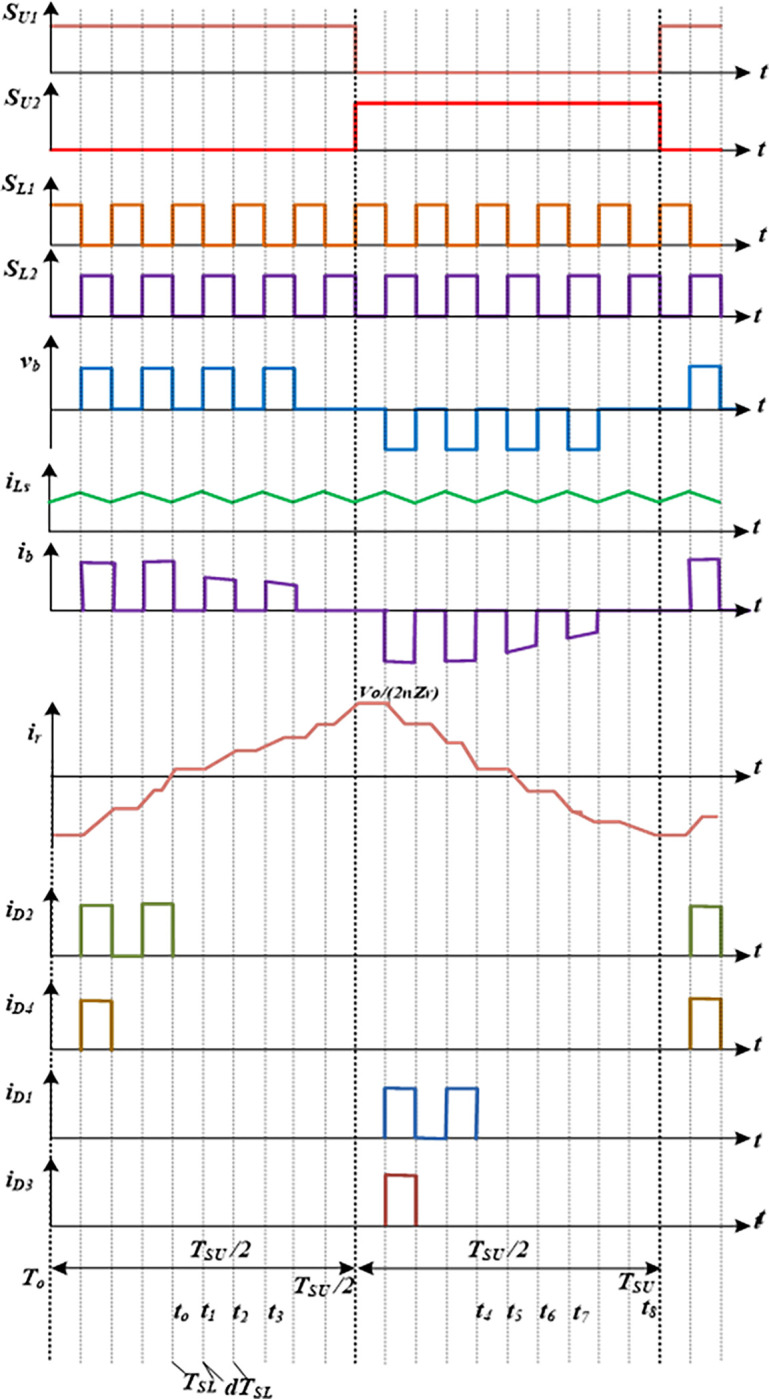
Key waveforms of the developed FBRC converter.

Fig 2 illustrates the main wave shapes of the proposed converter for two cascaded stages and one switching period. It shows the FB MOSFET switching signals, the voltage across the FB terminal (*v*_*b*_), current *i*_*b*_, the resonance current *i*_*r*_, and the cascaded multiplier diode current *I*_*D1*_*-I*_*D4*_. Due to the alternating behaviour of *i*_*b*_, the suggested topology’s CCM working approaches can be split into two parts: one for the +ve interval and another for the -ve interval, with corresponding time lengths [*T*_*o*_, *T*_*SU*_*/2*] and [*T*_*SU*_*/2*, *T*_*SU*_].

**Fig 3:**
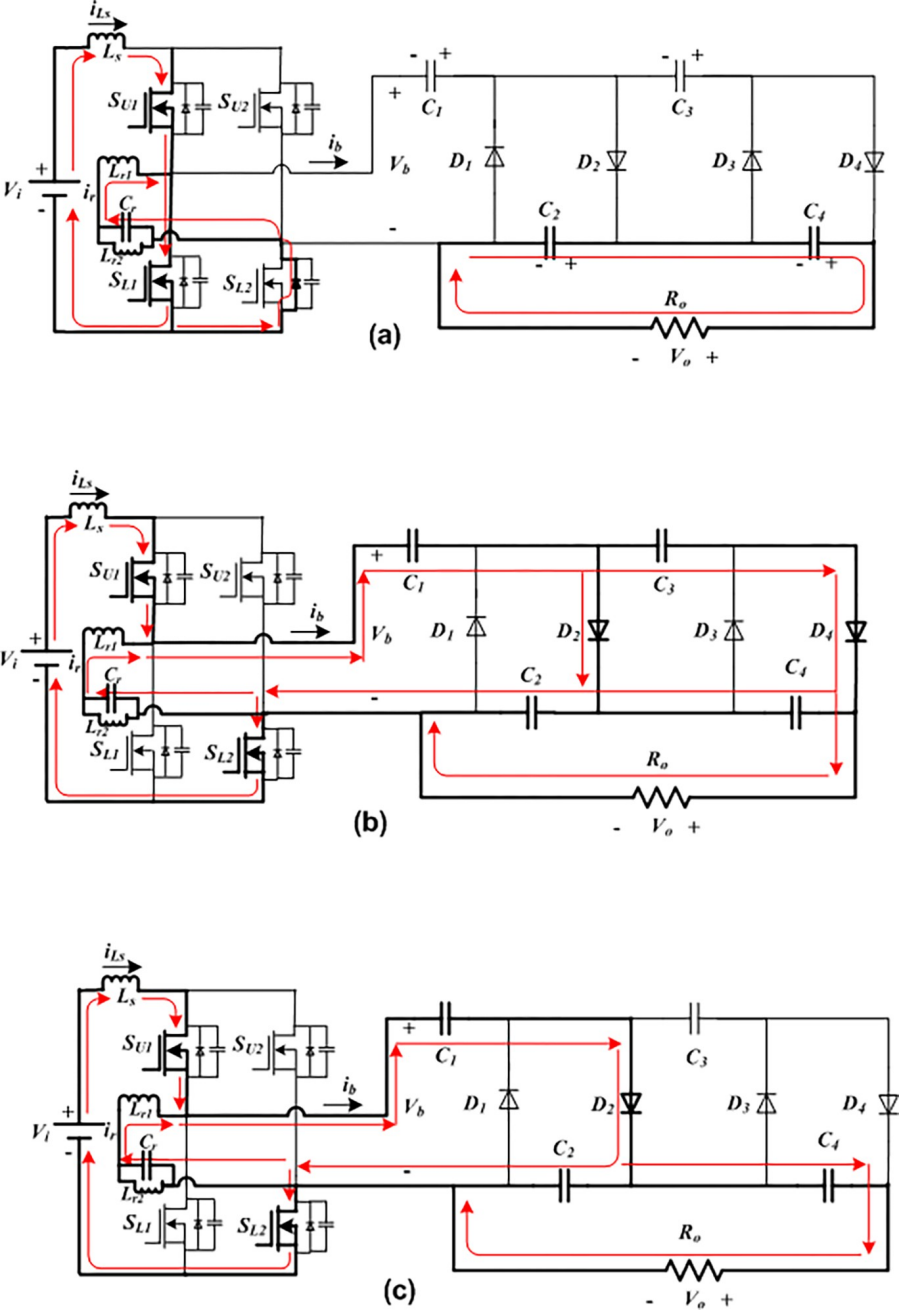
Current flow routes of the developed FBRC converter. (a) Operating Mode I. (b) Mode II-a. (c) Mode II-b.

**Fig 4:**
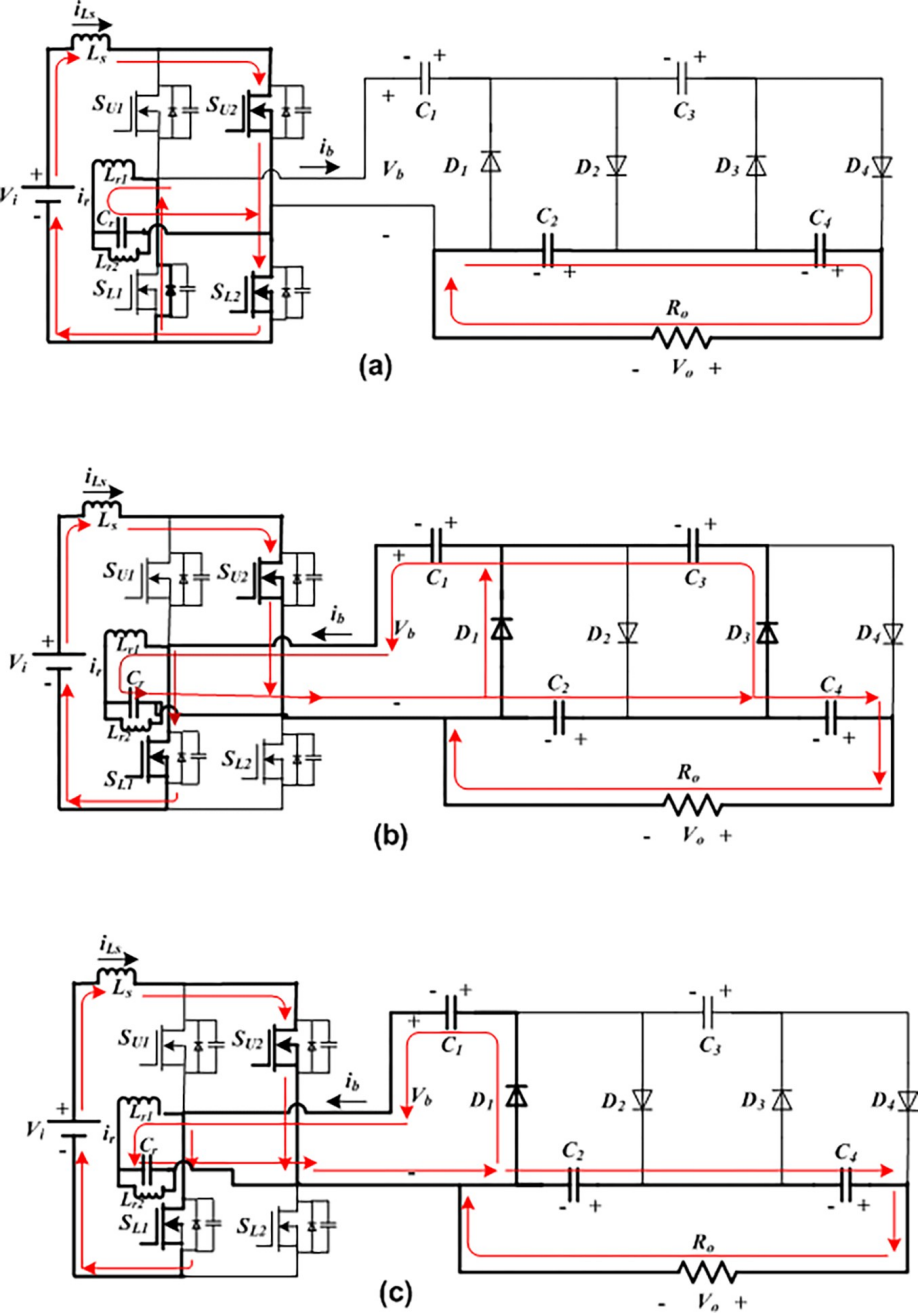
Current flow routes of the developed FBRC converter. (a)Mode III. (b)Mode IV-a. (c) Mode IV-b.

Only one diode conducts at order *D*_*4*_ followed by *D*_*2*_ in the positive half-cycle, and only one diode comes in conduction at order *D*_*3*_ followed by *D*_*1*_ in the other half-cycle. Furthermore, two operational modes, which are designated as Mode I and Mode II as displayed in Fig 3(A)–3(C) throughout this first half-cycle. Four distinct sections make up Mode II: Mode II-a, Mode II-b, Mode II-c and Mode II-d. As seen in Fig 4(A)–4(C), there are two working modes in the opposite interval of Mode III and Mode IV. Mode IV is further divided into four sub-modes, which are designated as Mode IV-a, Mode IV-b, Mode IV-c, and Mode IV-d. the *n*-stage diode-capacitor cascaded multipliers for which the proposed converter has undergone mathematical analysis have improved the converter’s applicability. Below is a detailed explanation of the circuit operation principles based on these operating modes.

Mode I (Fig 3(A)): In this stage, the high frequency switches *S*_*U1*_ is ON and *S*_*U2*_ is OFF, and all of the diodes of the voltage multiplier network are also switched OFF. At *t*_*o*_, the high-frequency gating signals are applied to *S*_*L1*_ and *S*_*L2*_, and *S*_*L1*_ is turned ON when ZVS conditions are met. Through conducting switches *S*_*U1*_ and *S*_*L1*,_ as well as the bypass diode of *S*_*L2*_, the boost inductor, resonant inductor, and capacitor are charged by the dc voltage source *V*_*i*_. while *C*_*3*_ and *C*_*1*_ stay floating, the capacitors *C*_*4*_ and *C*_*2*_ provide current to the output.

As seen in Fig 2, in this mode, the voltage across the FB terminal drops to zero, *v*_*b*_ = 0 throughout the interval *t*_*o*_ < *t* < *t*_*1*_. As a result, the resonant current is fixed as:

$$i_{Lr1}(t_{1})=i_{cr}(t_{1})=i_{r}(t_{1})=I_{r}$$ (1)

where *i*_*r*_ current passes through the resonant branch. On the other hand, the input boost inductor current can be found as follows:

$$\begin{array}{l} i_{Ls}(t_{1})=i_{SL1}(t_{1})-i_{r}(t_{1})\\ \;{=i_{SL1}(t_{1})-I}_{r}=\frac{{V_{i}-V}_{r}}{L_{s}}(t_{1}) \end{array}$$ (2)

where *i*_*SL1*_ is the current passes through the switch, *S*_*L1*_, and the resonant branch voltage is

$$V_{r}=v_{r1}+v_{cr}(v_{r2})$$ (3)

Mode II: In Mode II, at *t*_*1*_, *S*_*L2*_ is turned ON under the ZVS condition while *S*_*U1*_ stays ON. Concurrently, at *t*_*1*_, the upper switch in the lagging leg *S*_*U2*_ remains OFF, and *S*_*L1*_ is turned OFF under ZVS condition. Energy for the cascaded network is provided by the boost inductor, resonant branch, and dc input voltage source through various even group diodes (*D*_*2*_ and *D*_*4*_). Since diodes *D*_*4*_ and *D*_*2*_ conduct in Mode II-a, as seen in Fig 3(B), the capacitors *C*_*2*_ and *C*_*4*_ are charged by the FB current *i*_*b*_; and this current also discharges the capacitors *C*_*1*_ and *C*_*3*_. As can be seen in in Fig 3(C), in the subsequent Mode II-b, diode *D*_*2*_ conducts, which causes *i*_*b*_ to charge *C*_*2*_ and discharge *C*_*1*_; when *C*_*4*_ delivers to the load, *C*_*3*_ stays floating.

The voltage across the FB terminal at this step, *v*_*b*_ = *V*_*o*_/2*n* occurs at the time interval *t*_*1*_ < *t* < *t*_*2*_. The expression for the input inductor current *i*_*Ls*_ is:

$$\left(t_{2})=i_{b}(t_{2})-i_{r}(t_{2}\right)$$ (4)

$$i_{b}(t_{2})=i_{D2}(t_{2})+i_{D4}(t_{2})$$ (5)

iLs(t2)=Vi−vbLs(t2)=Vi−Vo2nLs(t2) (6)

where *V*_*i*_ and *v*_*b*_ stand for the voltages at the input dc source and FB output terminals *A* and *B*, respectively. The FB terminal voltage *v*_*b*_ can be calculated as follows:

$$v_{b}(t_{2})=v_{r1}(t_{2})+v_{Cr}(t_{2})=\frac{V_{o}}{2n}$$ (7)

where *v*_*r1*_ and *v*_*Cr*_ are the voltages across the resonant circuit inductor and capacitor respectively.

Therefore, the resonant voltage and current can be expressed as,

$$v_{r1}=L_{r1}\frac{di_{r}}{dt}$$ (8)

$$i_{r}=-\frac{V_{o}}{2{nZ}_{r}}\cos\omega_{r}(t_{2})$$ (9)

*i*_*r*_ is the resonant current, if the initial voltage $v_{r1(t_{1})}=0$, then Eq (8) becomes:

$$v_{r1}=\frac{V_{o}}{2n}\sin\omega_{r}(t_{2})$$ (10)

where the angular resonance frequency, $\omega_{r}=1/\sqrt{L_{r1}(C_{r}||L_{r2})}$ and resonant impedance, $Z_{r}=\sqrt{L_{r1}/(C_{r}||L_{r2})}$. Therefore, the voltage across the resonant capacitor, *v*_*Cr*_:

$$v_{Cr}=\frac{1}{C_{r}}\int_{t_{1}}^{t_{2}}i_{cr}\;dt$$ (11)

Combining Eqs (9) and (11),

$$v_{Cr}=-\frac{V_{o}}{{2n\omega_{r}Z}_{r}C_{r}}\sin\;\omega_{r}(t_{2})$$ (12)

From Eqs (5), (6) and (9),

ib(t2)=Vi−Vo2nLs(t2)−Vo2nZrcosωr(t2) (13)

As can be seen in Fig 2, there is an equal flow of current (*i*_*b*_) through diodes *D*_*2*_ and *D*_*4*_ throughout this time. Diode current may therefore be represented as follows using Eqs (5) and (13):

ID2(t2)=ID4(t2)=Vi−Vo2n2Ls(t2)−Vo4nZrcosωr(t2) (14)

Mode II-c (*t*_*2*_ < *t* < *t*_*3*_): during this interval Mode I will recur, meaning that switches *S*_*U1*_ and *S*_*L1*_ are turned ON, and the other switches (*S*_*U2*_ and *S*_*L2*_) and all the multiplier diodes are switched OFF. As a result, each current expression will be the same as what is mentioned in Mode I.

Mode II-d (*t*_*3*_ < *t* < *t*_*4*_): Mode II and Mode I will repeat one after the other during this period. Therefore, all of the current and voltage expressions should be the same as indicated above.

Mode III (*t*_*4*_ < *t* < *t*_*5*_): During this phase, switch *S*_*U2*_ and *S*_*L2*_ are turned on, and the opposite switches (*S*_*U1*_ and *S*_*L1*_) and all cascaded circuit diodes are turned off. The high-frequency operating switches *S*_*L1*_ and *S*_*L2*_ also function under ZVS conditions. The input dc voltage charges the boost input inductance (*L*_*s*_), the resonant inductor (*L*_*r*_), and the capacitor via the switches *S*_*U2*_ and *S*_*L2*_, and the *S*_*L1*_ switch’s bypass diode, respectively. As revealed in Fig 4(A), the bottom capacitors (*C*_*4*_ and *C*_*2*_) transfer energy to the load similarly to Mode I, while *C*_*3*_ and *C*_*1*_ stay in the floating position. In this mode, the resonant branch current (*i*_*r*_) is fixed during the interval *t*_*4*_ < *t* < *t*_*5*_, and the voltage across the FB terminal drops to zero, i.e., *v*_*b*_ = 0. Therefore, the boost inductor current can be calculated as:

$$i_{Ls}(t_{5})=i_{SL2}(t_{5})-i_{r}(t_{5}){=i_{SL2}(t_{5})-I}_{r}$$ (15)

where *i*_*SL1*_ is the current which flows through the switch, *S*_*L2*_.

Mode IV: *S*_*U2*_ and *S*_*L1*_ are switched ON, and *S*_*U1*_ and *S*_*L2*_ are turned off. The inductances and input dc source transfer energy to the diode-capacitor voltage multiplier circuit through the conducting of diodes *C*_*3*_ and *C*_*1*_. In Mode IV-a, as seen in Fig 4(B), the diodes *D*_*3*_ and *D*_*1*_ conduct, as a result, the FB current discharges *C*_*2*_ and charges *C*_*3*_ and *C*_*1*_; and load is driven by the current of *C*_*4*_. In the subsequent Mode IV-b, diode *D*_*1*_ is in conduction, which causes, *C*_*1*_ to be charged by *i*_*b*_, the load current is provided by *C*_*4*_ and *C*_*2*_, and when *C*_*3*_ stays in a floating state as shown in Fig 4(C).

During the interval *t*_*5*_ < *t* < *t*_*6*_, voltage across the FB terminal is *v*_*b*_ = *V*_*o*_/*n*, and the current *i*_*b*_ can be expressed as follows:

$$i_{Ls}(t_{2})=i_{b}(t_{2})-i_{r}(t_{2})$$ (16)

$$i_{b}(t_{6})=i_{D1}(t_{6})+i_{D3}(t_{6})$$ (17)

$$i_{Ls}(t_{6})=\frac{{V_{b}-v}_{i}}{L_{s}}(t_{6})$$ (18)

The FB terminal voltage, *v*_*b*_ can be determined as:

$$v_{b}(t_{6})=v_{r1}(t_{6})+v_{Cr}(t_{6})=\frac{V_{o}}{2n}$$ (19)

$$v_{r1}=L_{r1}\frac{di_{r}}{dt}$$ (20)

$$i_{r}=\frac{V_{o}}{{2nZ}_{r}}\cos\;\omega_{r}(t_{6})$$ (21)

*i*_*r*_ is the resonant current, then Eq (20) becomes:

$$v_{r1}=-\frac{V_{o}}{2n}\sin\;\omega_{r}(t_{6})$$ (22)

As a result, voltage across the resonant capacitor *v*_*Cr*_:

$$v_{Cr}=\frac{V_{o}}{2{n\omega_{r}Z}_{r}C_{r}}sin\omega_{r}(t_{6})$$ (23)

From Eqs (17), (18) and (21),

ib(t6)=−[Vi−Vo2nLs(t6)−Vo2nZrcosωr(t6)] (24)

According to Fig 4(B), *i*_*b*_, current flows through both diodes (*D*_*1*_ and *D*_*3*_) equally at this time. Therefore, from Eqs (17) and (24), diode current can be expressed as:

ID1(t6)=ID3(t6)=−[Vi−Vo2n2Ls(t6)−Vo4nZrcosωr(t6)] (25)

Mode IV-c (*t*_*6*_ < *t* < *t*_*7*_): Mode III will recur in this interval, with switch *S*_*U2*_ and *S*_*L2*_ turned ON; and the other switches (*S*_*U1*_ and *S*_*L1*_) as well as all of the voltage multiplier network diodes are switched OFF. Hence, every current expression will be the same as what Mode III states.

For *t*_*7*_ < *t* < *t*_*8*_, Mode IV-d: Modes IV and III will repeat in turn during this period. Therefore, every statement for current and voltage expression will ideally resemble those operating modes. The cycle will then begin once more after this.

## 3. Parameter design and selection

The full-bridge resonant cascaded (FBRC) converter’s dc voltage gain is contingent on the duty cycle, resonant frequency, and high-frequency switches switching frequency. The link between these variables is explained in this section, along with how different components are designed taking into account their stress and permitted input current and output voltage ripples. Furthermore, the range of ZVS operation of the FB switches has been explained along with the resonance circuit across the FB terminal that supplies the resonance current needed to accomplish ZVS has also been described. The resonance current required for the FB switches to achieve ZVS is provided by the resonant circuit in series with an inductor (*L*_*r*_) and a capacitor (*C*_*r*_) across the FB terminal. Furthermore, the converter can operate under ZVS state from full load to 25% load to this design.

### 3.1 DC voltage conversion ratio and duty cycle

**Fig 5:**
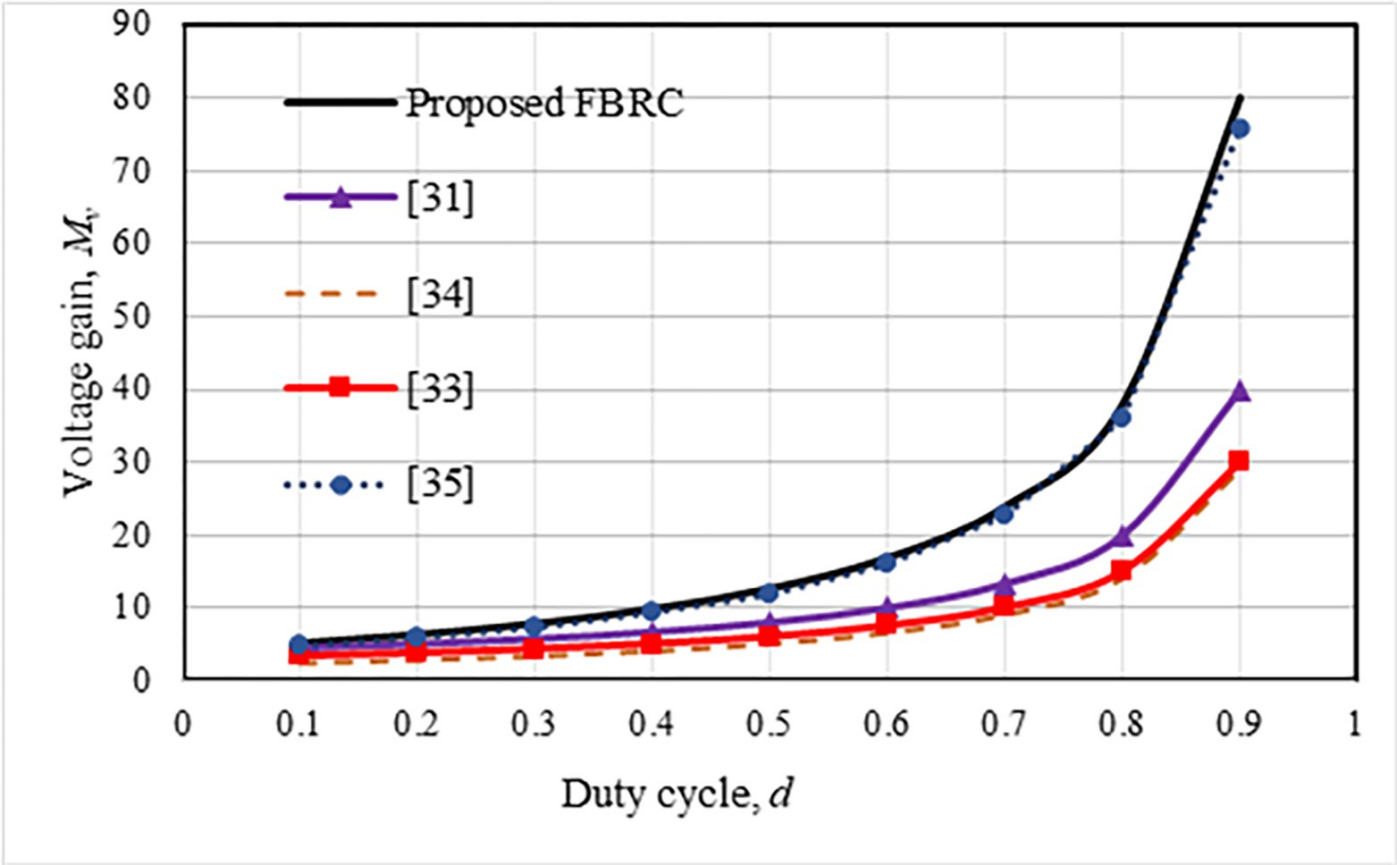
Voltage gain and duty cycle comparison.

**Fig 6:**
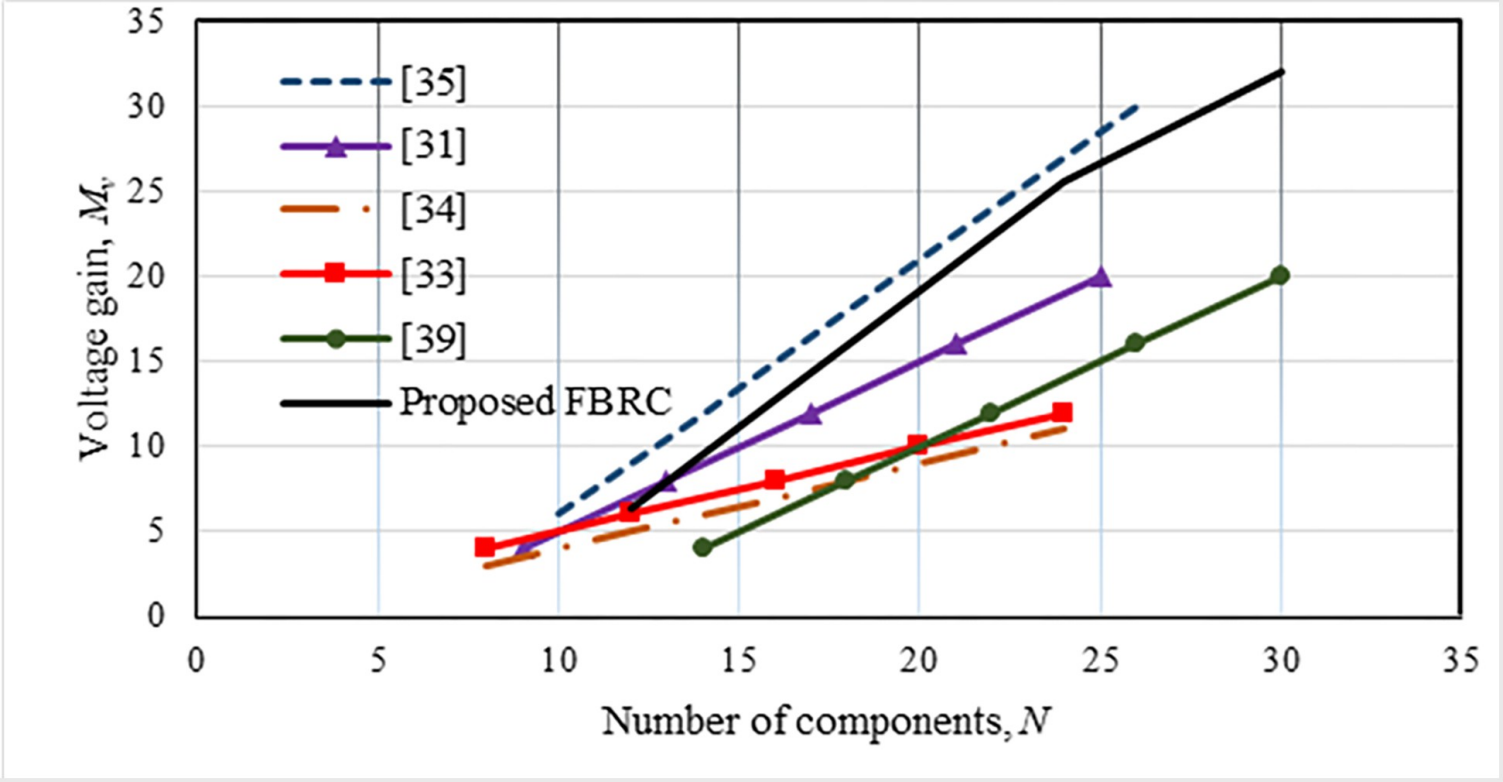
Comparison of voltage gain and number of major components.

The time durations (*t*_*o*_-*t*_*1*_) and (*t*_*1*_-*t*_*2*_) are precisely equivalent to the time spans *dT*_*SL*_ and (1-*d) T*_*SL*_, as seen in Fig 2, where *d* denotes duty ratio, and *T*_*SL*_ (1/*f*_*SL*_) is the switching signal period of switches *S*_*L1*_ and *S*_*L2*_. To express the dc voltage conversion ratio of this converter, one can substitute *dT*_*SL*_ and (1-*d)T*_*SL*_ for the time in Eqs (2) and (6), and then use the volt-second balance strategy upon the boost inductor (*L*_*s*_):

$$M_{V}=\frac{V_{0}}{V_{i}}=\frac{k_{r}+d}{1-d}2n$$ (26)

where *k*_*r*_ = *f*_*SL*_/*f*_*r*_ is the resonant constant, and *f*_*r*_ is the resonant frequency. In a resonant converter, the switching frequency should ideally match the resonance frequency or *k*_*r*_ = 1. In contrast, the switching frequency, *f*_*SL*_ is controlled to maintain regulated output voltage. Therefore, the resonant constant value is kept as *k*_*r*_ = 0.7~1.1 for ±10% variation of *f*_*SL*_. The voltage step-up gain of the suggested FBRC converter is compared with a few other converters described in the literature. It can be observed from Fig 5 that the voltage gain of this converter is higher than the other converters concerning the duty cycle. Also, although the component counts of this converter are slightly higher, the voltage step-up gain is higher compared to other converters except the converter described in [35], as shown in Fig 6.

### 3.2 Inductor selection

**Fig 7:**
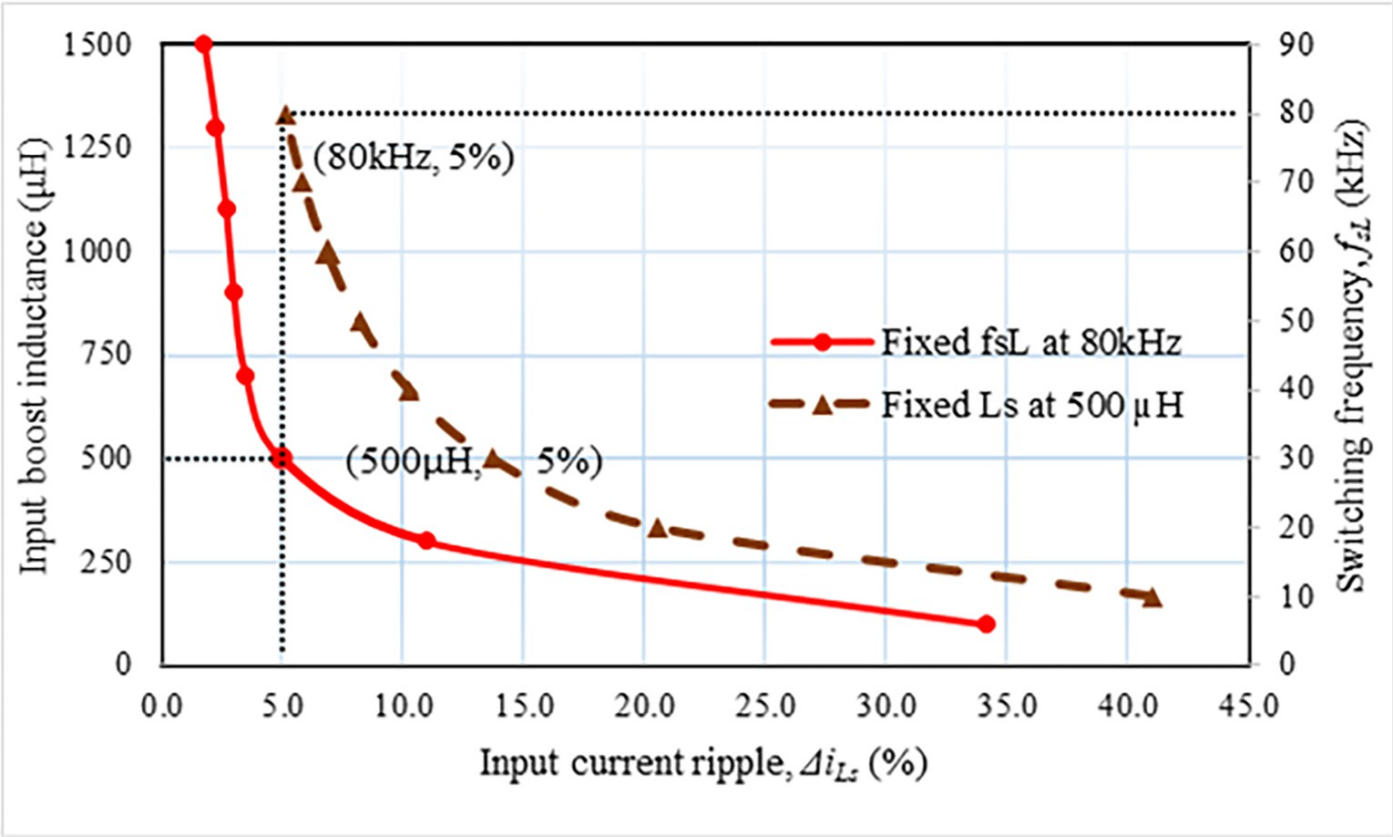
Input current ripple versus input inductance.

One important component of the suggested converter’s design that affects how much the input current ripples is the boost inductor. A fairly common nonlinear phenomenon is the PV array’s output changing the environmental conditions change. The suggested converter is made in a way that makes it ideal for a similar PV array. Nevertheless, ripple current has a massive impact on the PV array and significantly reduces its efficiency. The duty cycle must be raised as the *L*_*s*_ grows to control the output voltage and its ripple. Thus, the lowest possible input current ripple is taken into account together with the inductor’s size and cost. Boost input inductance (*L*_*s*_) can be determined as follows:

$$L_{2}=\frac{M_{V}.d}{I_{Ls.pk}.f_{SL}.\Delta i_{Ls}.pk.V_{o}}$$ (27)

where the input current ripple percentage and the maximum input current are designated by Δ*i*_*Ls*.*pk*_ and *I*_*Ls*.*pk*_, respectively. From Eq (27) it is realized that this ripple relies on the boost input inductance and the operating frequency (*f*_*SL*_) of *S*_*L1*_ and *S*_*L2*_ for a given voltage gain (*M*_*v*_) and duty cycle (*d*). The correlation between these factors is presented in Fig 7, where the solid line displays the boost input inductance versus input current’s ripple at the operating frequency, *f*_*SL*_ is 80 kHz, and the input ripple vs this frequency is revealed by the triangle marked (dashed) line at inductance of *L*_*s*_ = 500 μH. In all scenarios, the current ripples are the same and amount to 5%. Hence, the boost inductor for this converter is assumed to be 500 μH for *f*_*SL*_ and Δ*i*_*Ls*.*pk*_ are 80 kHz and 5%, respectively. The boost input inductance’s (*L*_*s*_) maximum stored energy can be computed as follows:

$$W_{Ls}=\frac{1}{2}L_{s}.{\left(\frac{V_{i}.d}{L_{s}.f_{SL}.\Delta i_{Ls}.pk}\right)}^{2}$$ (28)

The value can be computed as follows in the case of parallel inductance:

$$L_{p}=\frac{V_{b}.d}{I_{Lp.pk}.f_{SL}.\Delta i_{Lp}.pk}$$ (29)

where *I*_*Lp*.*pk*_ and Δ*i*_*Lp*.*pk*_ indicate as the parallel inductance’s peak current and its percentage of ripple. The parallel inductance’s (*L*_*p*_) maximum stored energy can also be computed as follows:

$$W_{Lp}=\frac{1}{2}L_{p}{.(\frac{V_{i}.d}{L_{p}.f_{SL.}\Delta i_{Lp}.pk})}^{2}$$ (30)

### 3.3 Capacitor selection and voltage stress

Except for the first capacitor, which has a voltage that is half of the others, all of the cascaded capacitors used in this design are suitably large and uniform in size, as was mentioned in the section above. Based on these presumptions, each capacitor’s voltage is as follows:

vcj={Vc2,forj=1Vc,forj=2,3…,N (31)

where the voltage of each capacitor, except the first (*C*_*1*_*)*, is *v*_*c*_, and *v*_*cj*_ represents the *j*th capacitor. From Fig 1, it is clear that voltage at the output terminal (*V*_*o*_) is equal to the voltage total of all bottom capacitors (*C*_*2*_, *C*_*4*_, …,) and can be determined as:

$$V_{o}={nV}_{c}$$ (32)

Combining Eqs (31) and (32) the voltage of each capacitor of the voltage multiplier circuit for the *n*-stage can be stated as follows:

vcj={Vo2n,forj=1Von,forj=2,3…N (33)

Therefore, according to Eq (33), the voltage multiplier capacitor’s maximum voltage stress is *V*_*o*.*pk*_/*2n*, and for the first capacitor, it is *V*_*o*.*pk*_/*n*, in which *V*_*o*.*pk*_ represents the output voltage’s peak value. The fifth row of Table 1 lists the voltage stresses for a single capacitor of this and alternative converter topologies. The value of the duty cycle and input dc voltage, as indicated in Table 1, are the only factors that affect the capacitor voltage stress for the recommended converter and the converter described in [31]. In contrast, the number of cascaded stages (*n*) of the other converters greatly influences the capacitor voltage stress.

**Table 1:** Comparison among conventional high voltage step-up ratio converters with the suggested converter.

Topology		Proposed	[31]	[34]	[35]	[33]	[39]
		(Fig 1)	(Fig 7)	(Fig 8)	(Fig 1)	(Fig 4)	(Fig 7)
**Voltage step-up gain**		$\frac{k_{r}+d}{1-d}2n$	$\frac{2n}{1-d}$	$\begin{array}{c} \frac{n+d}{1-d}\\ n=odd\\ \frac{n+1-d}{1-d}\\ n=even \end{array}$	$\frac{1+d}{1-d}2n$	$\frac{n+1}{1-d}$	$\frac{2n}{1-d}$
**Number of main devices**		5+4*n*	5+4*n*	4+4*n*	6+4*n*	4+4*n*	10+4*n*
**Voltage stress**	**Switch**	$\frac{V_{i}(k_{r}+d)}{1-d}$	$\frac{V_{i}}{1-d}$	$\frac{V_{i}}{1-d}$	$\frac{V_{i}(1+d)}{1-d}$	$\frac{V_{i}}{1-d}$	$\frac{V_{i}}{2(1-d)}$
	**Diode**	$\frac{{2V}_{i}(k_{r}+d)}{1-d}$	$\frac{2V_{i}}{1-d}$	$\frac{V_{i}}{1-d}$	$\frac{{2V}_{i}(1+d)}{1-d}$	$\frac{V_{i}}{1-d}$	$\frac{{2V}_{i}}{1-d}$
	**Capacitor**	$\begin{array}{c} V_{Cj}=\frac{V_{i}(k_{r}+d)}{1-d}\\ for\;j=1\\ =\frac{{2V}_{i}(k_{r}+d)}{1-d}\\ \mathrm{for}\;j=2,..,N \end{array}$	$\begin{array}{c} V_{Cj}=\frac{V_{i}}{1-d}\\ \mathrm{for}\;j=1\\ =\frac{{2V}_{i}}{1-d}\\ \mathrm{for}\;j=2,..,N \end{array}$	$V_{Cj1}{=V}_{Cj2}=\frac{jV_{i}}{1-d}$ for *j = 1*,..,*n*	$\begin{array}{c} V_{Cj}=\frac{V_{i}(1+d)}{1-d}\\ for\;j=1\\ =\frac{{2V}_{i}(1+d)}{1-d}\\ forj=2,..,N \end{array}$	$V_{Cj1}=\frac{jV_{i}}{1-d}$ for *j = 1*,..,*n* $V_{Cj2}=\frac{V_{i}}{1-d}$	$\frac{kV_{i}}{2(1-d)}$

N.B: *k* indicates the turn ratio of the transformer.

**Fig 8:**
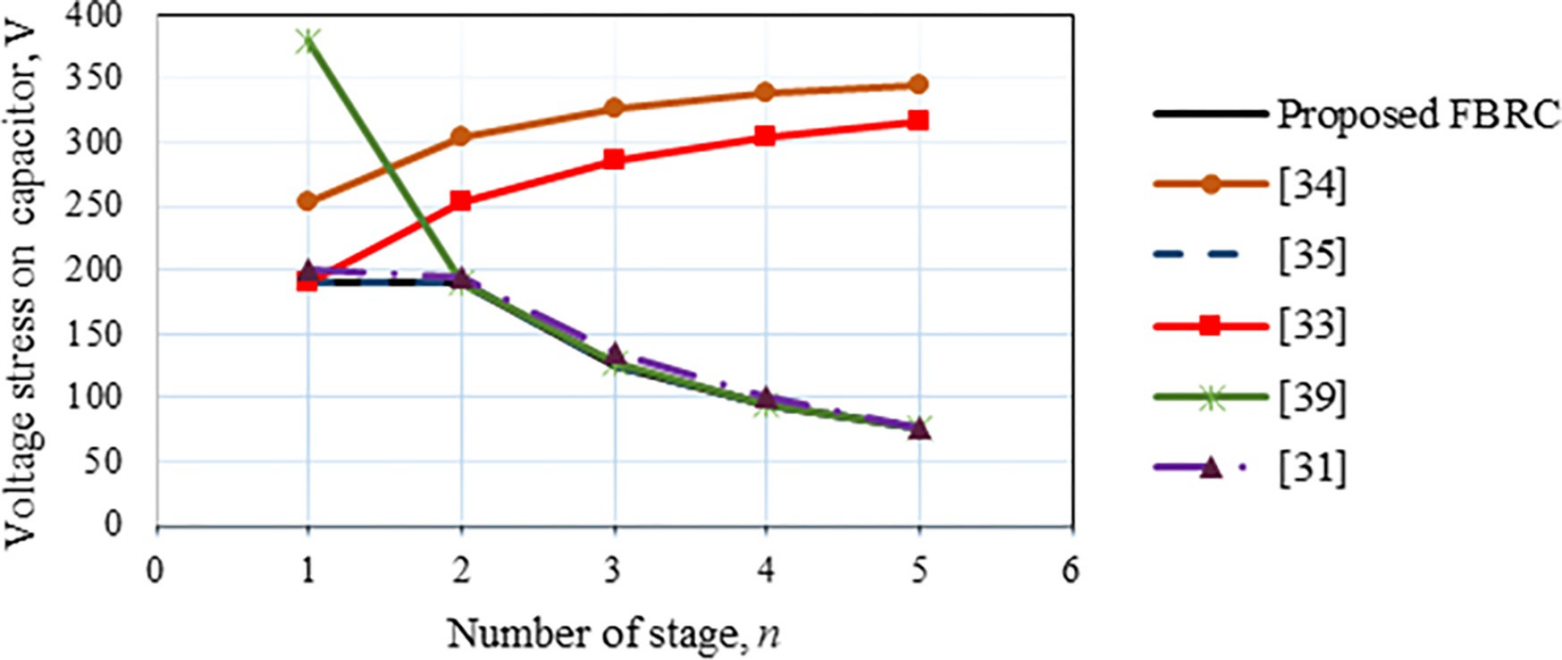
Comparison of the voltage stresses on the capacitor of the proposed and other converters.

Fig 8 illustrates voltage strains on the capacitor for this converter and a few others at a constant duty ratio, *d* = 0.5 and a constant output voltage, *V*_*o*_ = 380 V. Therefore, it can be seen from the voltage step-up gain equation of these architectures listed in Table 1 that the number of stages increases, the required input voltage declines. For instance, if there are two stages, (*n* = 2), and constant *d* = 0.5 and constant *V*_*o*_ = 380 V, the proposed converter input voltage is 31.67 V, whereas the converter reported in [34] requires a higher voltage, which is 76 V. As a result, the suggested topology has a lower capacitor voltage stress than the later one and also lower compared to the other converters up to the number of voltage multiplier stages, *n* = 2 as displayed in Fig 8.

The voltage across the capacitors about duty ratio can be stated as follows from Eqs (33) and (26) by considering the ideal value of *k*_*r*_ = 1:

vcj={Vi(1+d)1−d,forj=12Vi(1+d)1−d,forj=2,3…,N (34)

**Fig 9:**
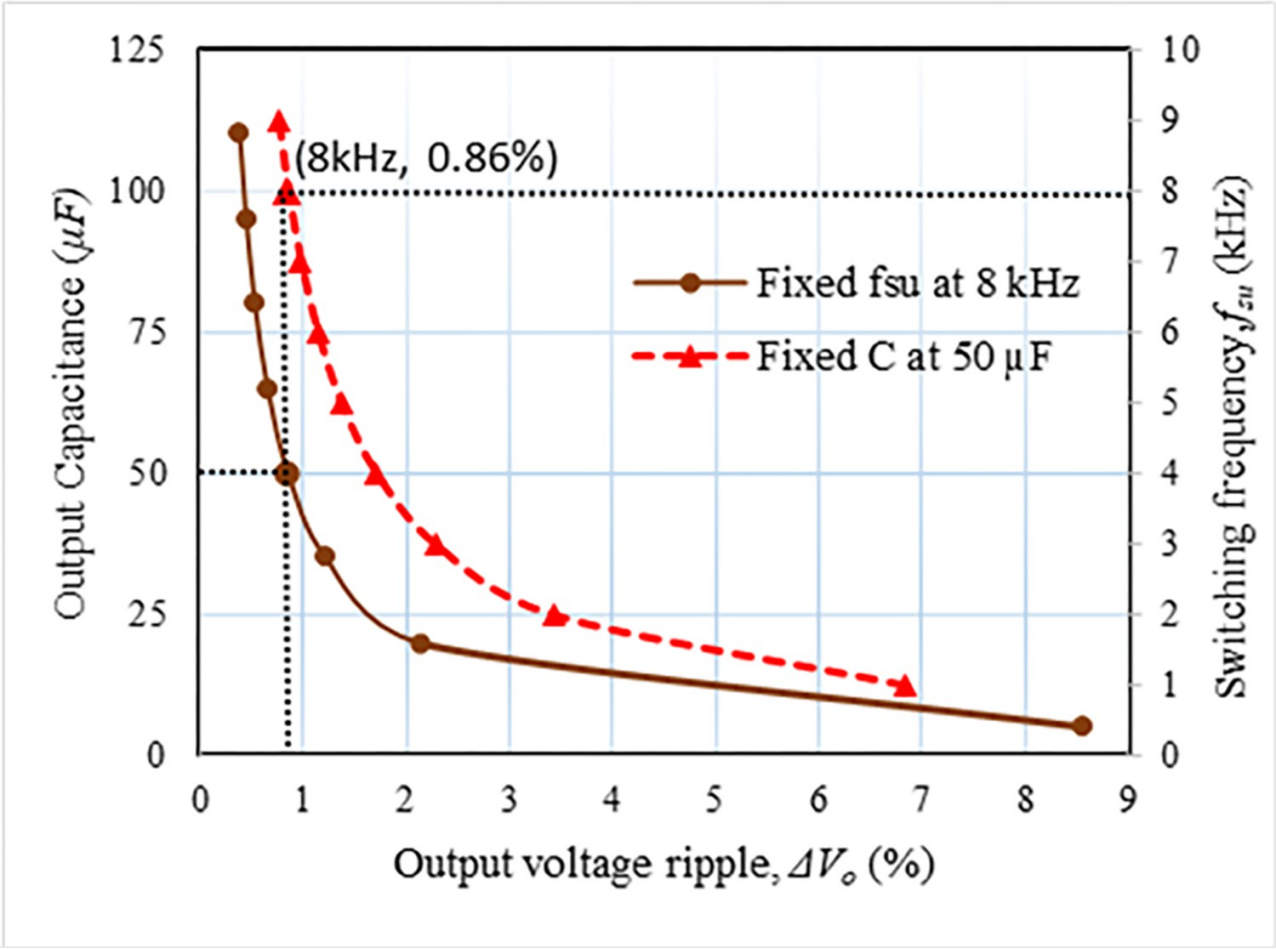
Capacitor voltage and output voltage ripple.

**Fig 10:**
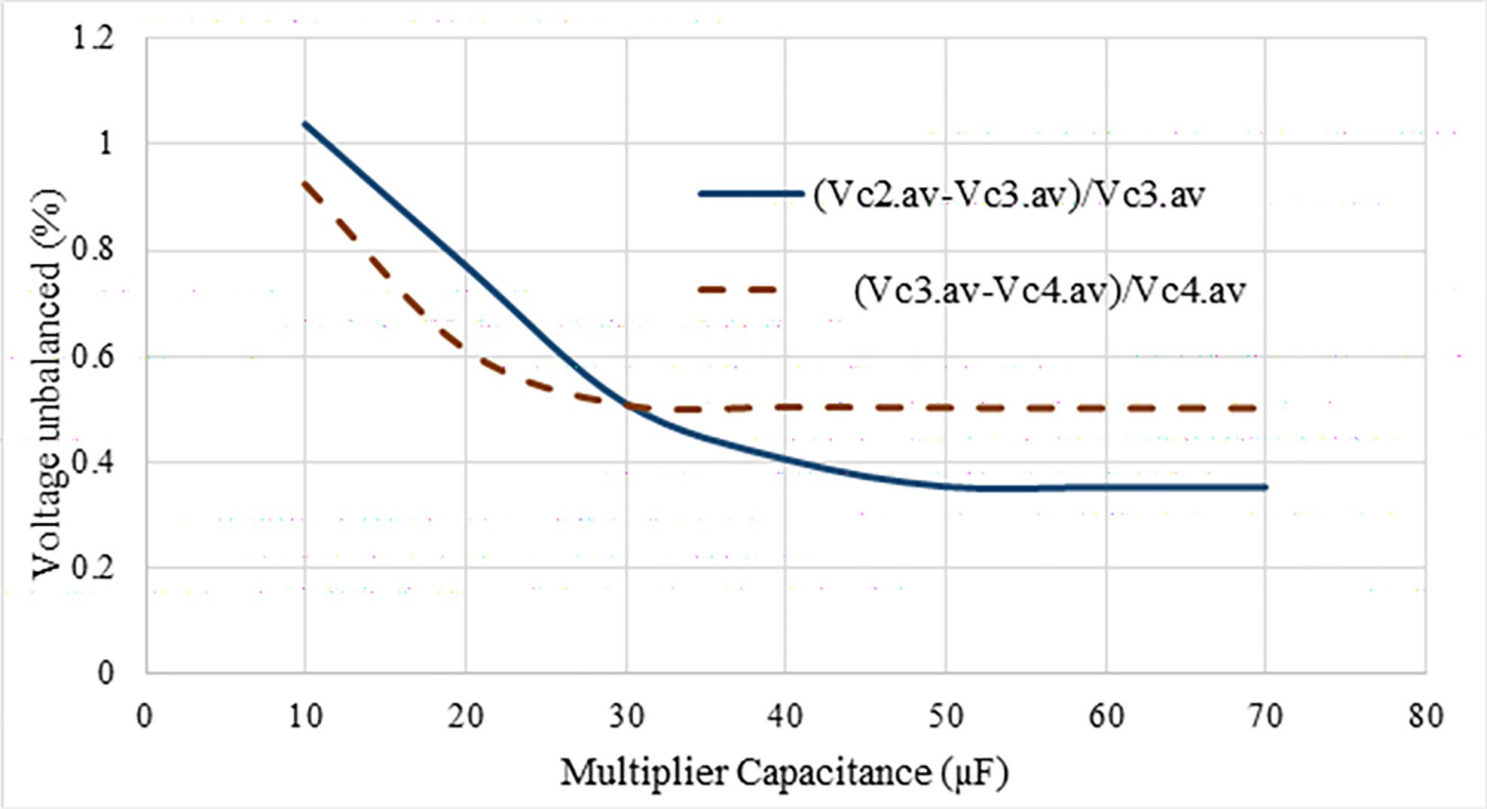
Voltage unbalanced ripple on the capacitor.

As can be observed from Eq (34), unlike the other converter topologies described in the literature, the individual capacitor voltage of this converter fluctuates with variations in the *V*_*i*_ and *d* rather than the number of voltage multiplier stages. Although all capacitor voltages are equal in theory, it is impossible to ignore the voltage drops and ripples that occur when a capacitor is loaded. The ripple voltage of each specific capacitor is as follows, according to the current-fed investigation [36], which is not as much of intricate as the corresponding voltage-fed study [37, 38]:

$$\Delta V_{cj}=\frac{I_{o.av.}T_{SU}}{C}\left(\frac{N-j+1}{2}\right)\;for\;j=1,2,3,\ldots ,N$$ (35)

where *T*_*SU*_ and *I*_*o*.*av*_ stand for the time duration of the alternating frequency and the average output current, respectively. The ripple of the output voltage for a given current output and the number of voltage multiplier stages can be observed from Eq (35) depending on the gate signal frequency (*f*_*SU*_) of the upper two switches (*S*_*U1*_ and *S*_*U2*_) as well as the foot side capacitance. Fig 9 represents the correlations between these factors. The solid line in Fig 9 indicates the capacitance versus ripple voltage at a given operating frequency, *f*_*SU*_ is 9 kHz. Additionally, the operating frequency, *f*_*SU*_ versus ripple is displayed by the triangle marked dashed line at the given capacitance value, *C* = 50 μF. The ripple of the output voltage should be the same 0.76% in both scenarios. Therefore, for switching frequency, *f*_*SU*_, is 9 kHz and *Δv*_*C*,_ is 0.76%, the capacitance is chosen as 50 μF for this suggested converter. There is a little bit of voltage unbalance in the multiplier capacitor as revealed in Fig 10. At capacitance 50 μF, voltage imbalance is found as 0.2%.

The stored energy in capacitance can be given by:

$$w_{cj}=\frac{1}{2}C_{j}v_{cj}^{2}$$ (36)

By submitting Eqs (34) and (35) into Eq (36),

wcj=Io.av.Tsa2ΔVcj(N−j+12){(Vi(1+d)1−d)2,forj=1(2Vi(1+d)1−d)2,forj=2,3…,N (37)

### 3.4 Switch’s voltage and current stresses

**Fig 11:**
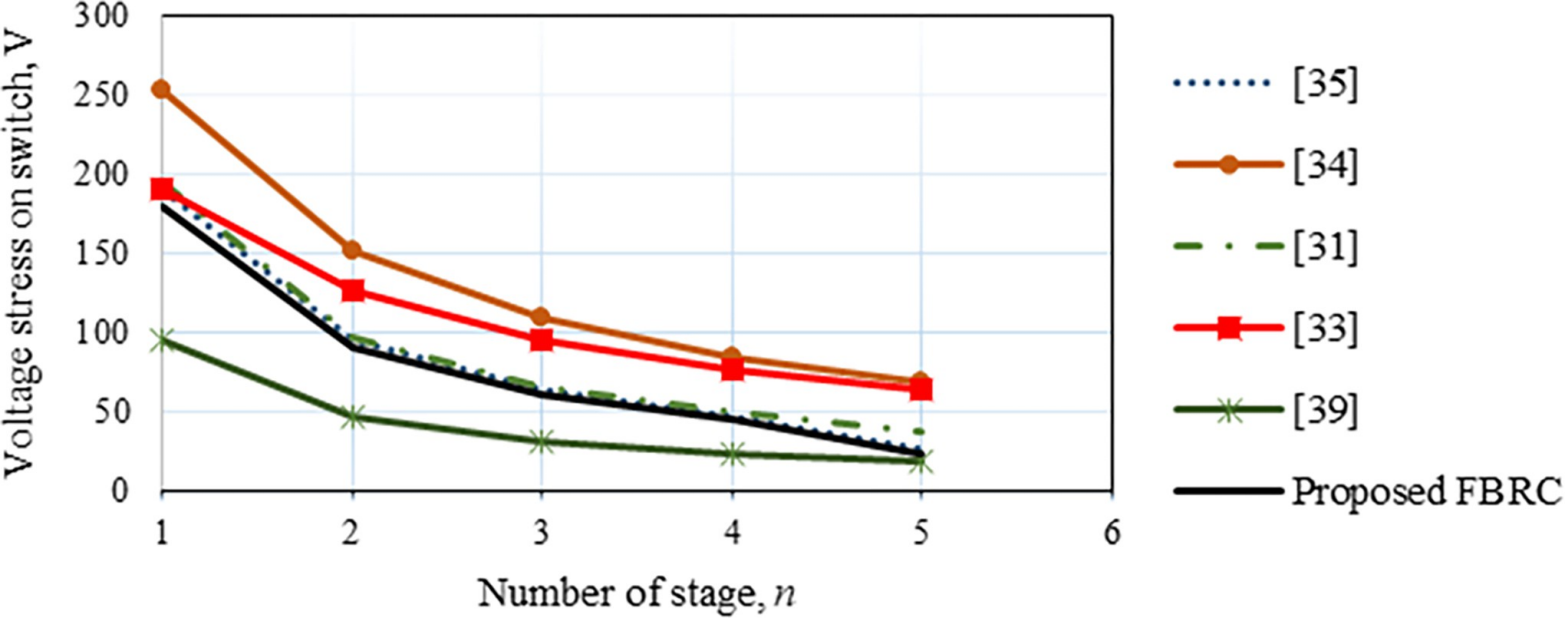
Voltage stress on switch of the proposed FBRC converter.

The highest voltage stress of the diode is twice that of the gate switches which is *V*_*o*.*pk*_*/2n*, and the extreme stress of current is *I*_*b*.*pk*_*/n*, where *I*_*b*.*pk*_ is the peak current input of the voltage multiplier circuit. Two diodes are on at once. The switch’s voltage stress is represented in Fig 11. It is lower for this suggested converter than others except the converter reported in [39].

Table 2 represents the performance such as the output power, efficiency, voltage ripple, current ripple, voltage gain, and duty ratio of some boost-type dc-dc converters suitable for renewable energy (e.g., PV, FC, etc.) applications compared with the suggested converter. The voltage gain is higher for the converter in [40] than the others, whereas the ON time of the switch(s) is kept lower for the proposed converter. In addition, the measured efficiency is higher than all other converters. Furthermore, the converter in [41], offers the least output voltage ripple; however, it suffers from poor efficiency at the rated power. The output voltage and input current ripples are much higher in the converter [18] than that of the developed converter. Moreover, the proposed converter’s output power, voltage gain, efficiency, and ripples are better than the converter described in [35].

**Table 2:** Comparison among the output power, duty cycle, voltage gain, voltage and current ripples, and efficiency of different dc-dc converters.

Parameter	[42]	[40]	[41]	[35]	[18]	Proposed
Topology	Fig 6(A)	Fig 1	Fig 7	Fig 1	Fig 10(B)	Fig 1
Output power, *P*_*o*_(W)	100	225	120	250	300	500
Duty ratio, *d*	0.65	0.65	0.5	0.5	0.8	0.5
Voltage gain, *M*_*v*_	8.44	11.11	9	8.44	--	10
Output voltage ripple, *ΔV*_*o*_(%)	1	<1	0.6	0.86	4.1	0.76
Input current ripple, *ΔI*_*i*_(%)	20	--	--	6.8	43	5
Efficiency, *η*(%)	94.86	93.2	90	94.5	93.7	95.8

### 3.5 Condition and range for ZVS of the switches

As seen in Fig 3, the ZVS current of switch *S*_*L2*_ is the difference between the bridge current (*i*_*b*_) and the auxiliary resonant branch current (*i*_*r*_) at *t*_*2*_. On the other hand, as Fig 3(E) illustrates, the ZVS current of switch *S*_*L1*_ is the result of a combination of the bridge current (*i*_*b*_) and the auxiliary resonant branch current (*i*_*r*_) at *t*_*6*_. Given that the operating frequencies of these two switches are the same (*t*_1_ = *t*_2_ = *t*_6_). As a result, these two currents have the same RMS value. Therefore,

ISL.ZVS=Ib+Vo2nZrcosωrt1=Vi−Vo2nLs+Vo2nZrcosωrt1 (38)

The following requirements must be met for *S*_*L1*_ and *S*_*L2*_’s ZVS to switch on and off:

$$\frac{1}{2}{(L_{r1}+L_{r2}).I_{SL.ZVS}}^{2}>\frac{1}{2}(C_{sL1}+C_{sL2}){\left(\frac{V_{o}}{2n}\right)}^{2}$$ (39)

It is clear from Fig 3 that the RMS current (*I*_*SU*.*ZVS*_) flowing through the upper two switches (*S*_*U1*_ and *S*_*U2*_) has the same magnitude as *I*_*SL*.*ZVS*_. However, current flow through these switches for a longer period than *t*_*1*_ is approximately 8.89 times longer (8.89 is the frequency ratio of *f*_*SL*_ and *f*_*SU*_ for this study). Consequently, the lower frequency switches (*S*_*U1*_ and *S*_*U2*_) likewise operate securely under ZVS conditions across the whole operational range.

## 4. Control technique

**Fig 12:**
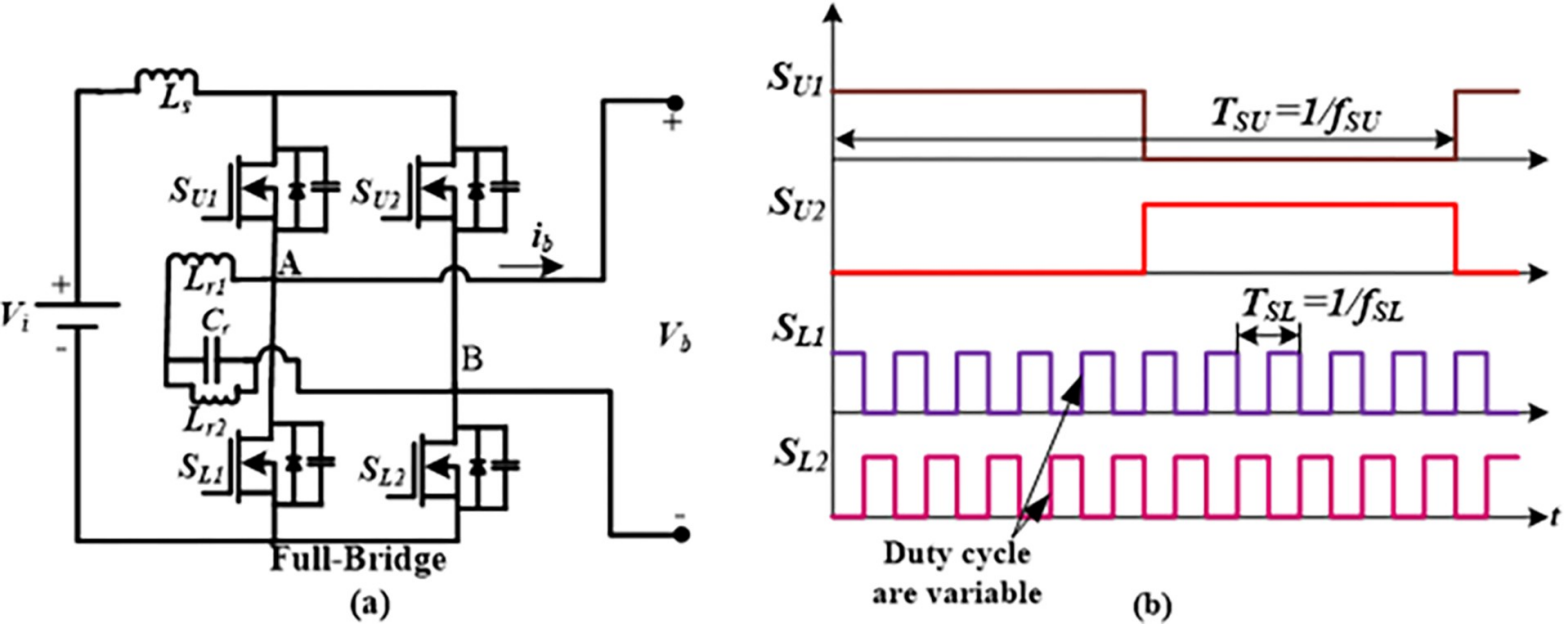
(a) Full-bridge module. (b) Control gate signals of the full-bridge module of the proposed converter.

This section describes the control technique of the proposed converter in brief. The proposed converter operates similarly to the classic boost converter, except for the alternating voltage (*v*_*b*_) and current (*i*_*b*_) generated across the full-bridge terminal. Due to the cost-effectiveness, fast processing speed, and user-friendly application, the DSP chip has generated control signals for the designed converter. The suggested converters utilize a two-independent-frequency variable-duty cycle PWM modulation technique to generate the gate signals. The FB module has four switches, *S*_*U1*_, *S*_*U2*_, *S*_*L1*_, and *S*_*L2*_, as depicted in Fig 12(A). *S*_*U1*_ (*S*_*L1*_) and *S*_*U2*_ (*S*_*L2*_) work in a complementary manner, with *S*_*U1*_’s switching frequency denoted as *f*_*SU*_ and *S*_*L1*_’s switching frequency marked as *f*_*SL*_, as shown in Fig 12(B). *f*_*SU*_ and *f*_*SL*_ are designated as the alternating frequency and modulating frequency, respectively, for the sake of ease. Ideally, choosing these two frequencies as high as possible is necessary to minimize the usage of lower values of passive components (capacitor and inductor) in the circuit. This study achieves the desired output by maintaining a significantly lower frequency for *f*_*SU*_ compared to *f*_*SL*_. The output voltage *V*_*o*_ is controlled by adjusting the duty ratio of *f*_*SL*_, while *f*_*SU*_ determines the ripple of *V*_*o*_. Furthermore, the *f*_*SL*_ to *f*_*SU*_ ratio must be an odd number (an integer or a fraction). Specifically, if the ratio (*f*_*SL*_/*f*_*SU*_) is not an even integer *F*, the voltage across the FB (*v*_*b*_) will be unidirectional instead of alternating. This is because two switches of the same FB lag, *S*_*U1*_ and *S*_*L1*_ or *S*_*U2*_ and *S*_*L2*_, will turn on simultaneously at the transition of *f*_*SU*_.

### 4.1 PID controller design

The state-space averaging methodology is commonly employed to design linear controllers for dc-dc converters due to its ability to integrate the advantages of both state-space and averaging methods. It uses the inductor current and capacitor voltage as separate variables. This converter’s continuous time-domain transfer function can be expressed using the state-space averaging technique as follows:

$$\frac{{\hat{v}}_{o}(s)}{\hat{d}(s)}=\frac{V_{o}}{dL_{e}C_{o}}\left[\frac{s^{2}\frac{L_{e}R_{c}C_{o}}{R_{o}}+sR_{c}(C_{o}-L_{e})+\frac{R_{c}}{R_{o}}+1}{s^{2}+s(\frac{\left(R_{c}/d\right)}{L_{e}}+\frac{1}{{C_{o}R}_{o}})+\frac{\left(1/d^{2})+(R_{c}/d\right)}{L_{e}{C_{o}R}_{o}}+\frac{1}{{L_{e}C}_{o}}}\right]$$ (40)

where *V*_*o*_, *R*_*o*_ and *C*_*o*_ represent the output voltage, load resistance, and output filter capacitance, respectively. The duty cycle, represented by *d*, measures the ratio of the on-time to the total time of a periodic signal. *R*_*C*_ and *L*_*C*_ represent the equivalent series resistance (ESR) and equivalent series inductance (ESL) of *C*_*o*_ and *C* components, respectively. Here, *C* equals *C*_*1*_ and *C*_*3*_, while *C*_*o*_ equals the parallel combination of *C*_*2*_ and *C*_*4*_. To simplify the analysis, the equivalent series inductance (ESL) of the capacitor and the equivalent series resistance (ESR) of the inductor are ignored. ${\hat{v}}_{o}(s)$ and $\hat{d}(s)$ represent the minor fluctuations in the output voltage and duty cycle, respectively, and *L*_*e*_ = *L*_*C*_/(1−*d*)^2^. The parameters’ values can be found in Tables 3 and 4, as well as in their respective data sheets. Upon submitting these values, the control to output transfer function at the nominal operating point is determined as:

$$\frac{{\hat{v}}_{o}(s)}{\hat{d}(s)}=\frac{3.503*10^{-6}s^{2}+2.117*10^{-5}s+1.217*10^{3}}{4.128*10^{-4}s^{2}+3.614*10^{-2}s+4.231*10^{2}}$$ (41)

**Table 3:** The proposed converter prototype’s specifications.

Parameters	Value
Output power, *P*_*o*_	500 W
Output voltage, *V*_*o*_	400 V
DC input voltage, *V*_*i*_	40~80 V
Alternating frequency, *f*_*sa*_	9 kHz
Modulation frequency, *f*_*sm*_	80 kHz
No. of stage, *n*	2

**Table 4:** Description of suggested converter prototype’s components.

Components and symbol	Value / Part no.
MOSFET, *S*_*U1*_, *S*_*U2*_, *S*_*L1*,_ *S*_*L2*_	C3M0120090D (SiC)
Diode, *D*_*1*_ *~ D*_*4*_	IDH10S120 (SiC)
Capacitor, *C*_*1*_ *~ C*_*4*_	4× 50 μF
Boost inductor, *L*_*s*_	500 μH
Parallel inductor, *L*_*r1*,_ *L*_*r2*_	100 μH, 50 μH
Parallel capacitor, *C*_*r*_	6.5 μF
Voltage sensor	LEM LV 25-P
Gate driver IC	HCPL-3120
Controller, DSP	TMS320F28335

The PID controller, a lead-lag compensator, is extensively employed in feedback control systems. Thus, the PID controller is selected to regulate the proposed converters. The PID controller can be defined as:

$$m(t)=K_{P}e(t)+K_{I}\int_{0}^{t}e(t)dt+K_{D}\frac{de(t)}{dt}$$ (42)

where *e(t)* and *m(t)* represent the input and output of the compensator, respectively. The Laplace transform of the transfer function described in Eq (42) can be expressed as:

$$G_{c}(s)=\frac{M(s)}{E(s)}=K_{P}+\frac{K_{I}}{s}+K_{D}s$$ (43)

This continuous time domain transfer function is converted to a discrete-time domain to implement in the DSP controller. Therefore, the digital transfer function of the PID controller can be derived as follows:

$$G_{c}(z)=K_{P}+\frac{K_{I}Tz}{z-1}+\frac{K_{D}(z-1)}{Tz}$$ (44)

**Fig 13:**
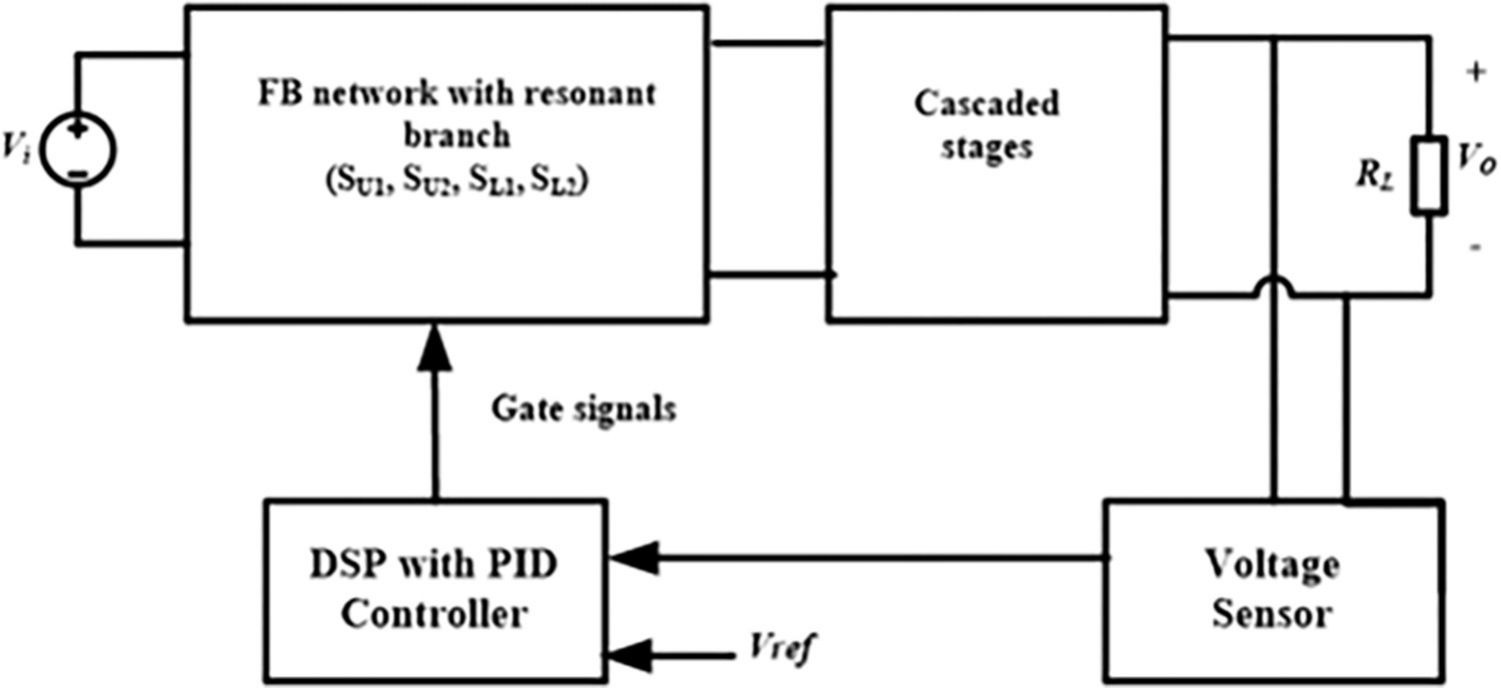
Block diagram of the control technique of the proposed converter.

The z-domain transfer function of the PID controller in Eq (44) needs to be transformed into a difference equation to generate a new duty cycle for the digital PID controller using DSP. The difference equation can be expressed as:

$$u[k]=K_{P}e[k]+K_{I}T\sum_{i=0}^{k}e[i]+\frac{K_{D}}{T}\{e[k]-e[k-1]\}$$ (45)

where *u[k]* and *e[k]* represent the controller output and output error, respectively, for the *k*th sample. The various parameter values are located in the experiment and data sheets. Fig 13 displays the block diagram of the control technique used in this converter. The voltage sensor measures the output voltage and transmits it to the DSP module. The digital signal processor (DSP) produces switching gate signals for the FB MOSFET by comparing the output voltage (*V*_*o*_) with the reference voltage (*V*_*ref*_), using the PID controller technique.

## 5. Experimental outcomes

**Fig 14:**
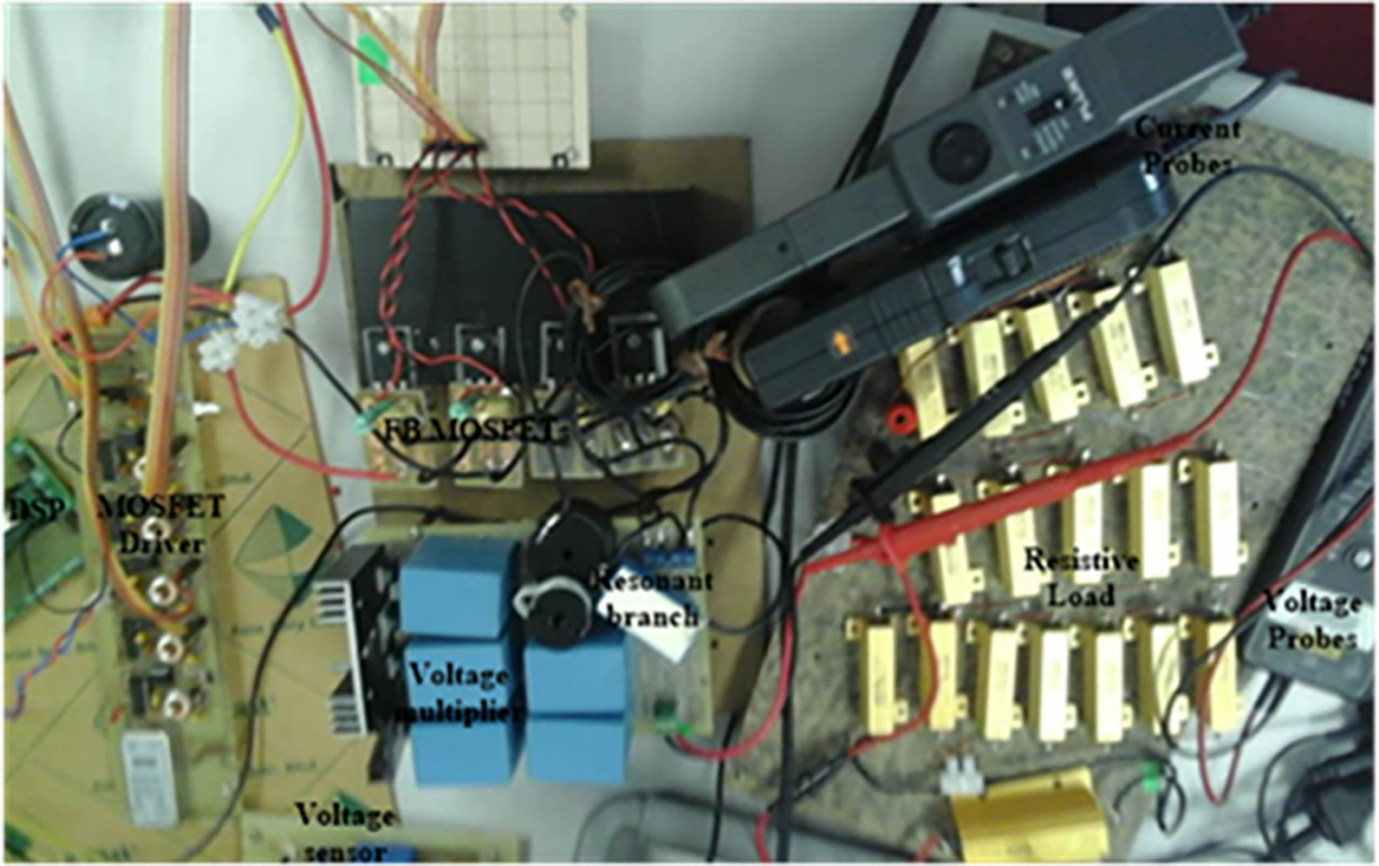
FBRC converter hardware implementation.

To validate the theoretical analysis for the developed FB resonant cascaded (FBRC) dc-dc converter, a 500 W laboratory prototype of two cascaded stages is implemented. The figure of the experimental setup indicating all apparatus is displayed in Fig 14.

To get the intended output voltage, *V*_*o*_ = 400 V, a broad range of input voltages (40 ~ 80 V) is applied. The duty ratio of the gate switching frequency (*f*_*SL*_) is adjusted between 0.45 ~ 0.6. The Texas Instrument TMS320F28335 controller generates and controls the switching signals. Tables 3 and 4 list the component values following the design process and the various parameters employed in the developed converter.

### 5.1 Result analysis of FBRC converter

**Fig 15:**
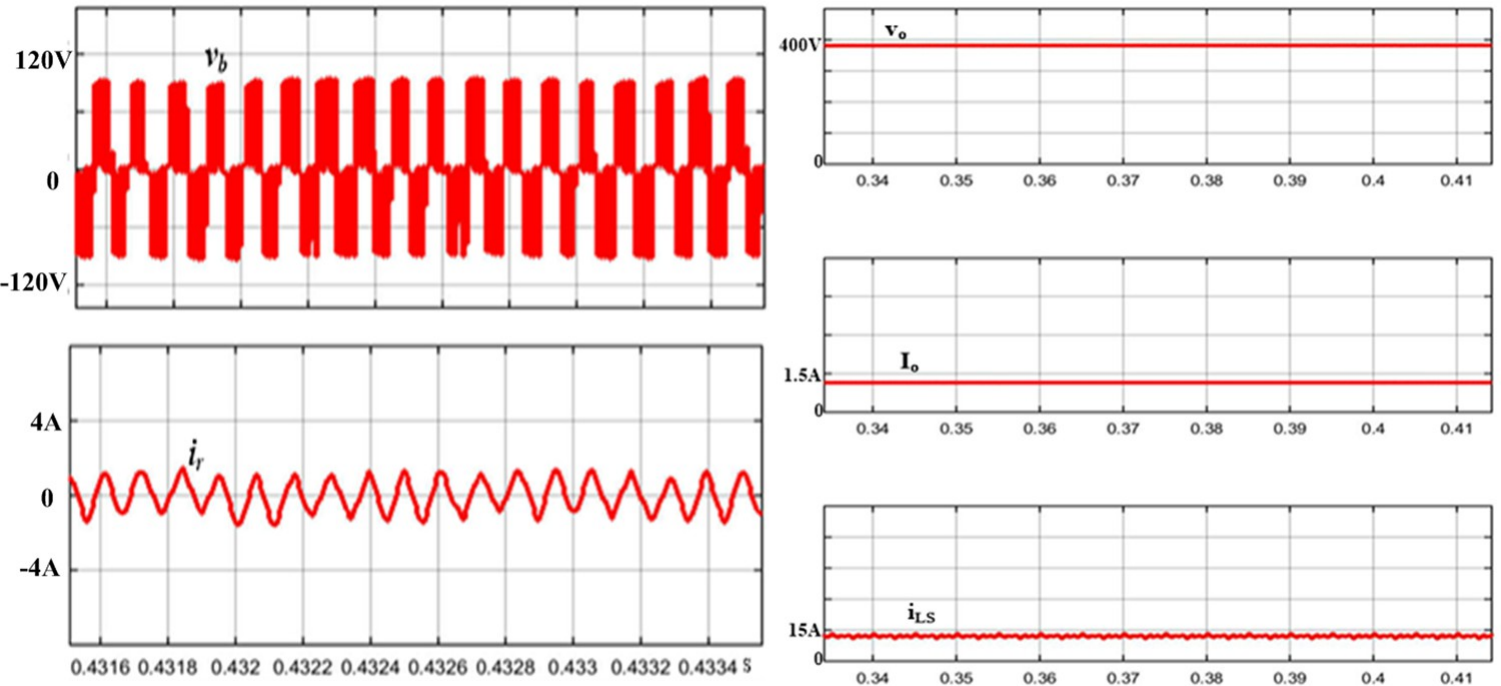
Simulation waves of *V*_*b*_ and *i*_*r*_, output voltage and current, *V*_*o*_ and *I*_*o*_, and input current *i*_*Ls*_.

The simulation results of the suggested Full-bridge resonant cascaded (FBRC) converter in the steady-state mode are illustrated in Fig 15. The gate signals of the FB switches *S*_*SU1*_, *S*_*SU2*_, *S*_*SL1*,_ and *S*_*SL2*_, where the first two of these have an alternating frequency *f*_*SU*_ (9 kHz), while the second two use a higher frequency *f*_*SL*_ (80 kHz). Fig 15 also demonstrates the simulation outcomes of the FB circuit terminal voltage (*v*_*b*_), and resonant current (*i*_*r*_) along with the output voltage (*V*_*o*_), output current (*I*_*o*_), and input current (*i*_*LS*_).

**Fig 16:**
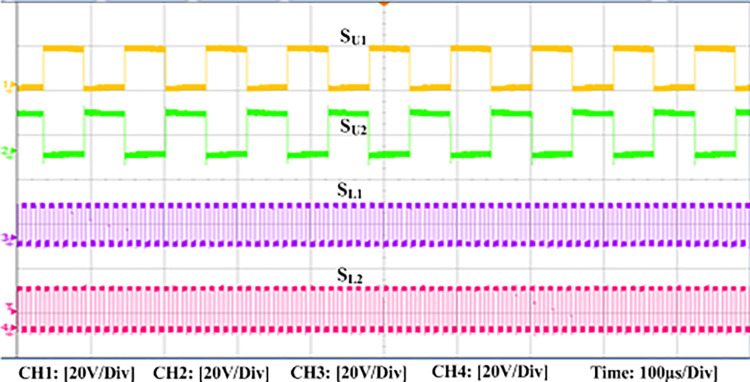
Experimental switching signals of the four FB MOSFET gates.

**Fig 17:**
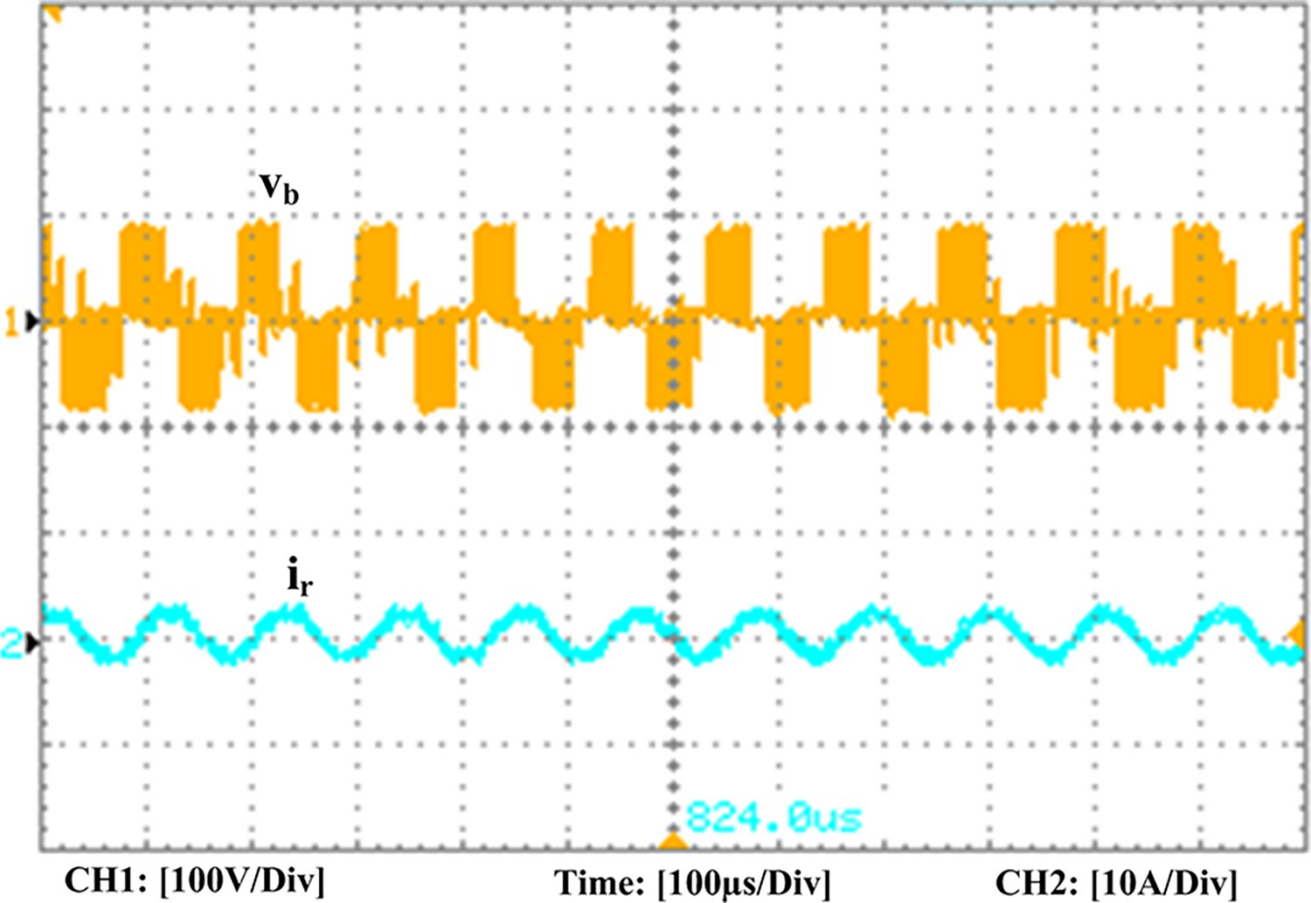
Experimental results of FB voltage (*v*_*b*_) and resonant current (*i*_*r*_).

**Fig 18:**
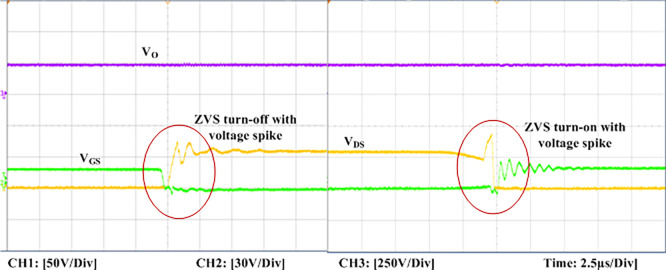
ZVS turn-off and turn-on with voltage spike at *f*_*SL*_(88kHz) > *f*_*r*_(80kHz) of the switch *S*_*L1*_.

**Fig 19:**
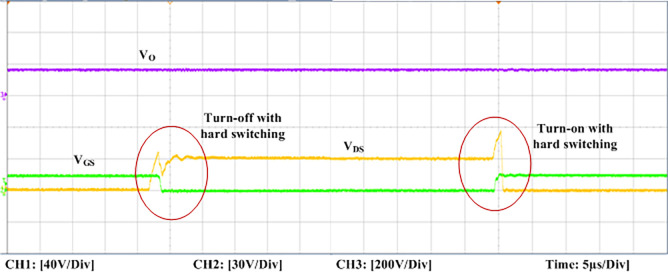
Turn-off and turn-on with hard switching at *f*_*SL*_(38 kHz) < *f*_*r*_*/2* of the switch *S*_*L1*_.

**Fig 20:**
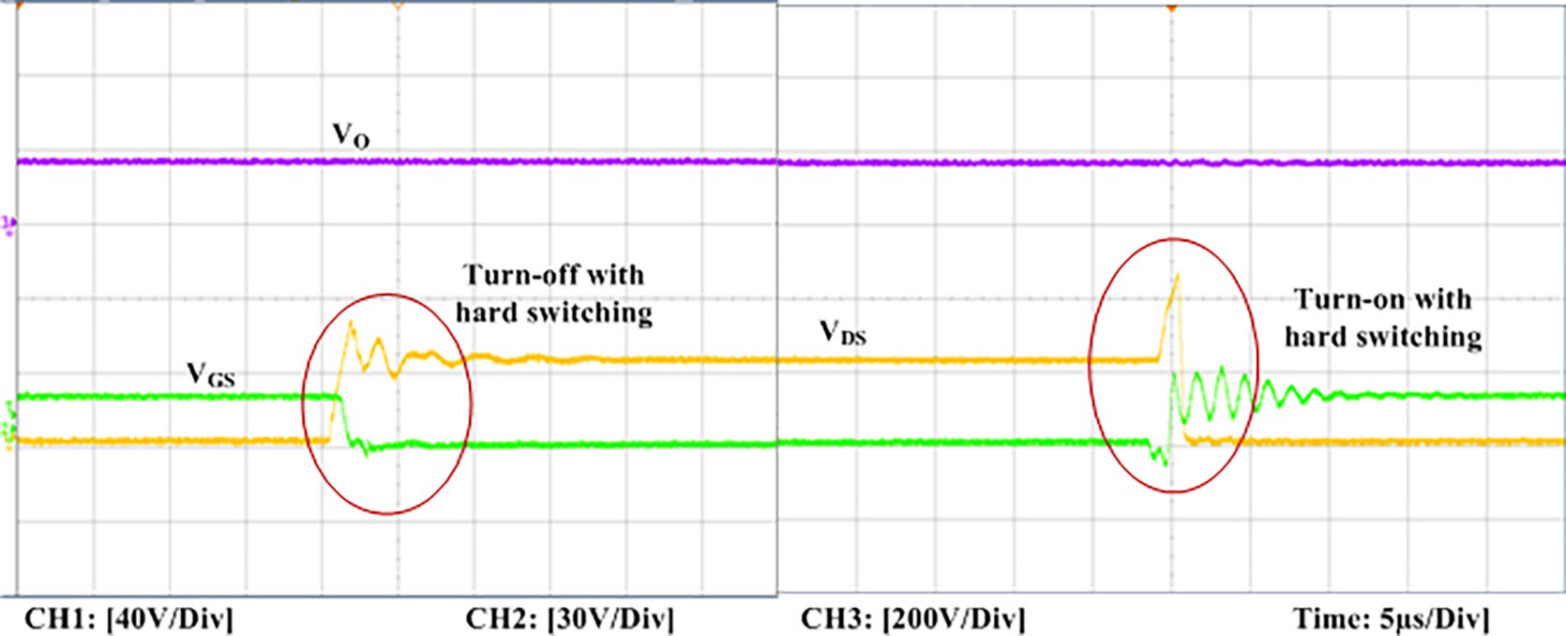
Turn-off and turn-on with hard switching at *f*_*r*_*/2* < *f*_*SL*_(50 kHz) < *f*_*r*_ of the switch *S*_*L1*_.

**Fig 21:**
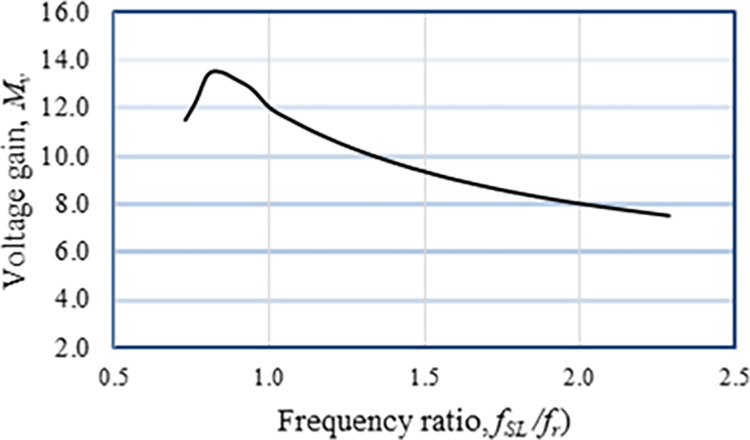
Voltage gain versus frequency ratio.

**Fig 22:**
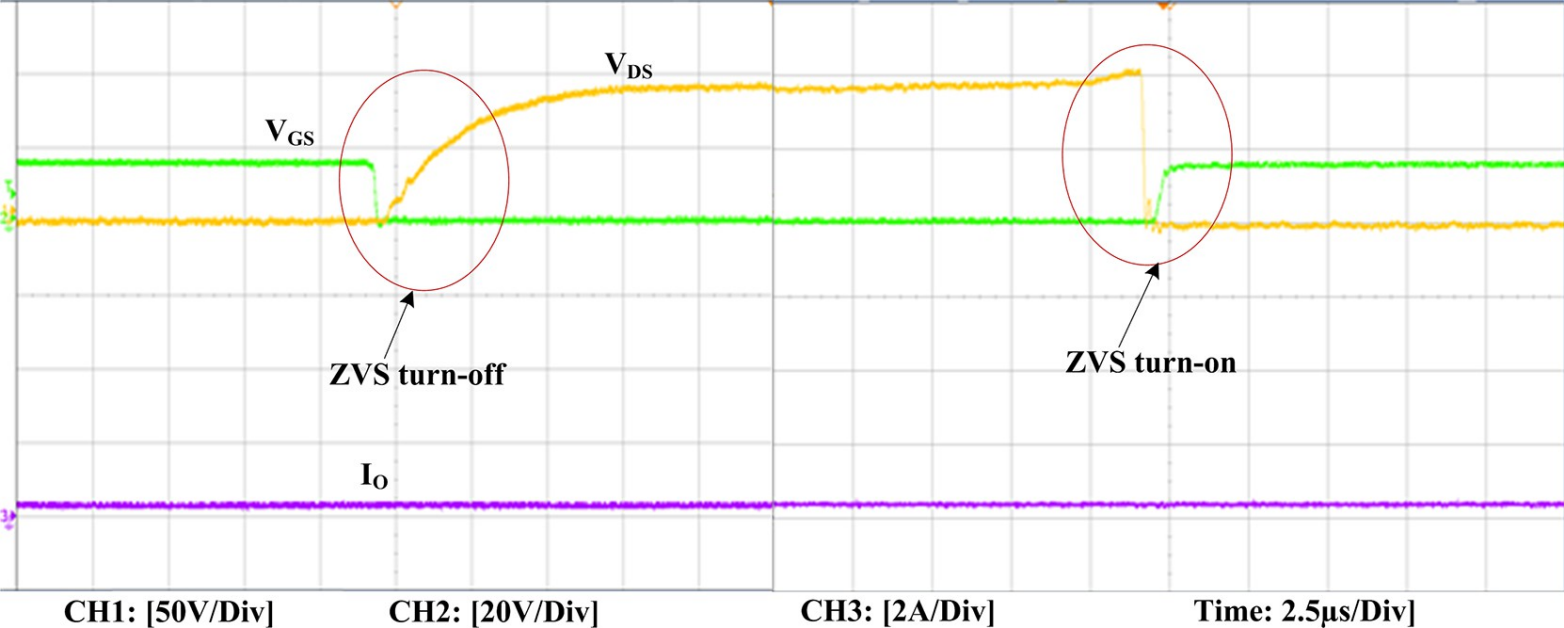
ZVS turn-off and turn-on operation at 25% load of *S*_*L1*_.

**Fig 23:**
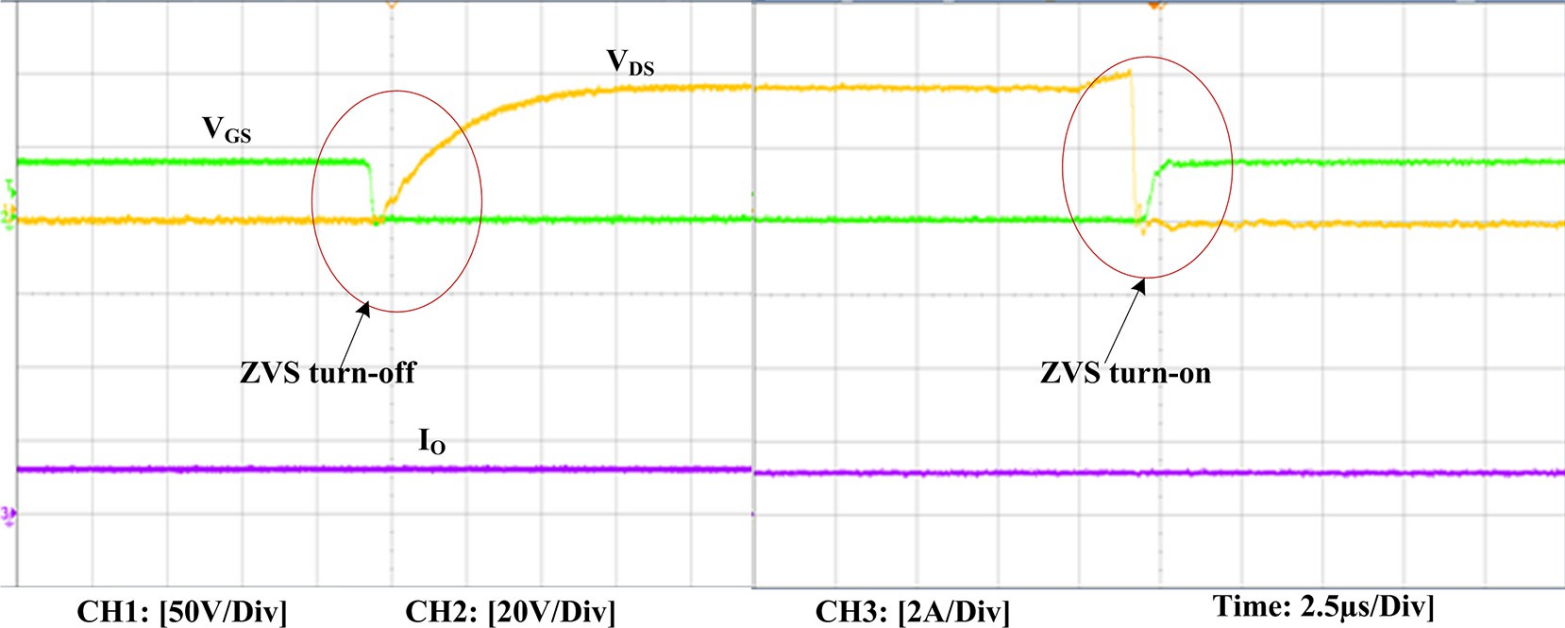
ZVS turn-off and turn-on operation at 100% load of *S*_*L1*_.

**Fig 24:**
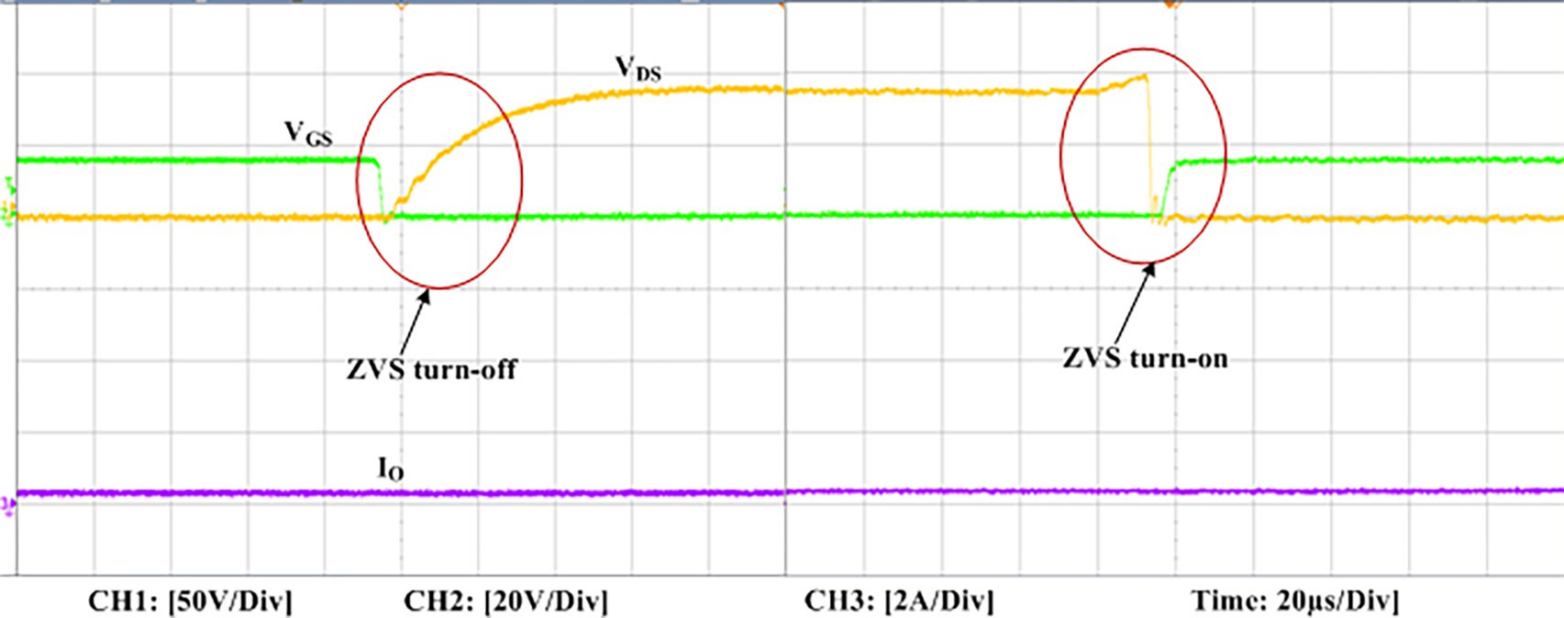
ZVS turn-off and turn-on operation at 25% load of *S*_*U1*_.

**Fig 25:**
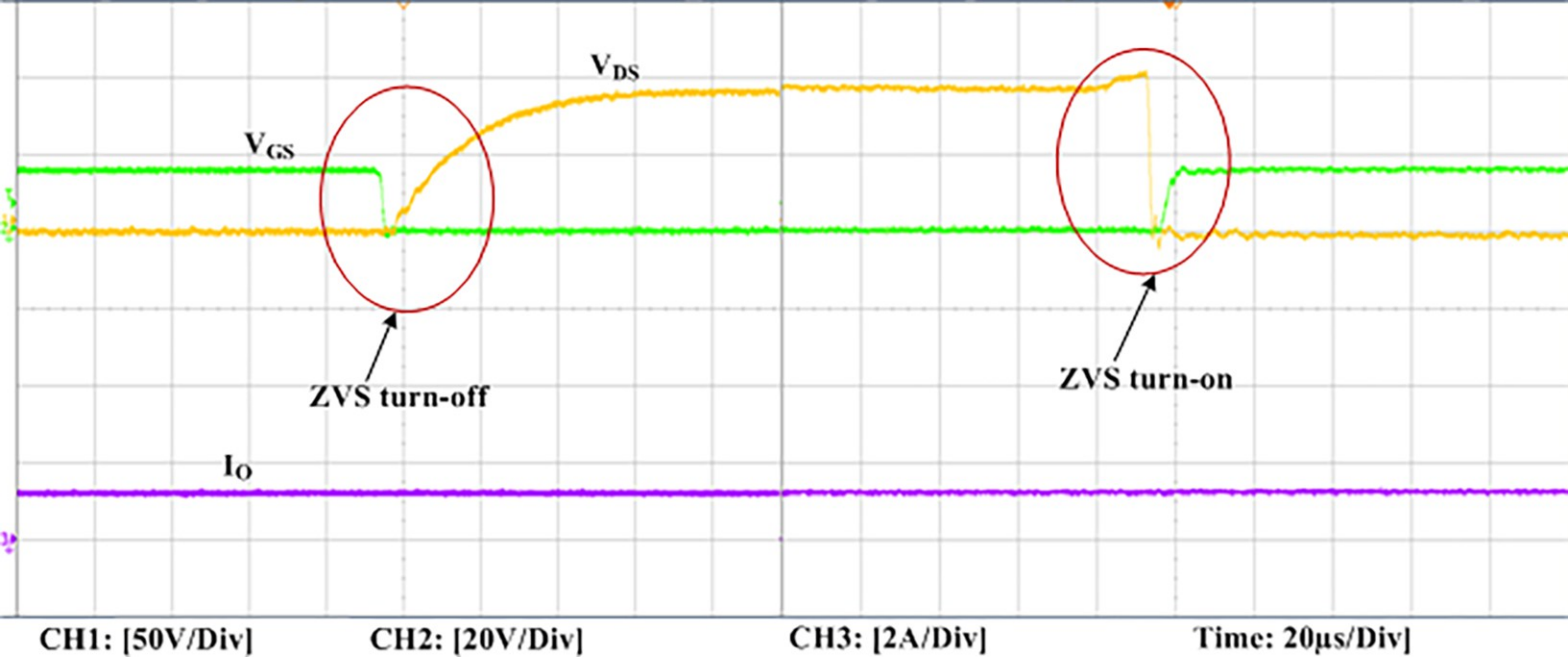
ZVS turn-off and turn-on operation at 100% load of S_U1_.

**Fig 26:**
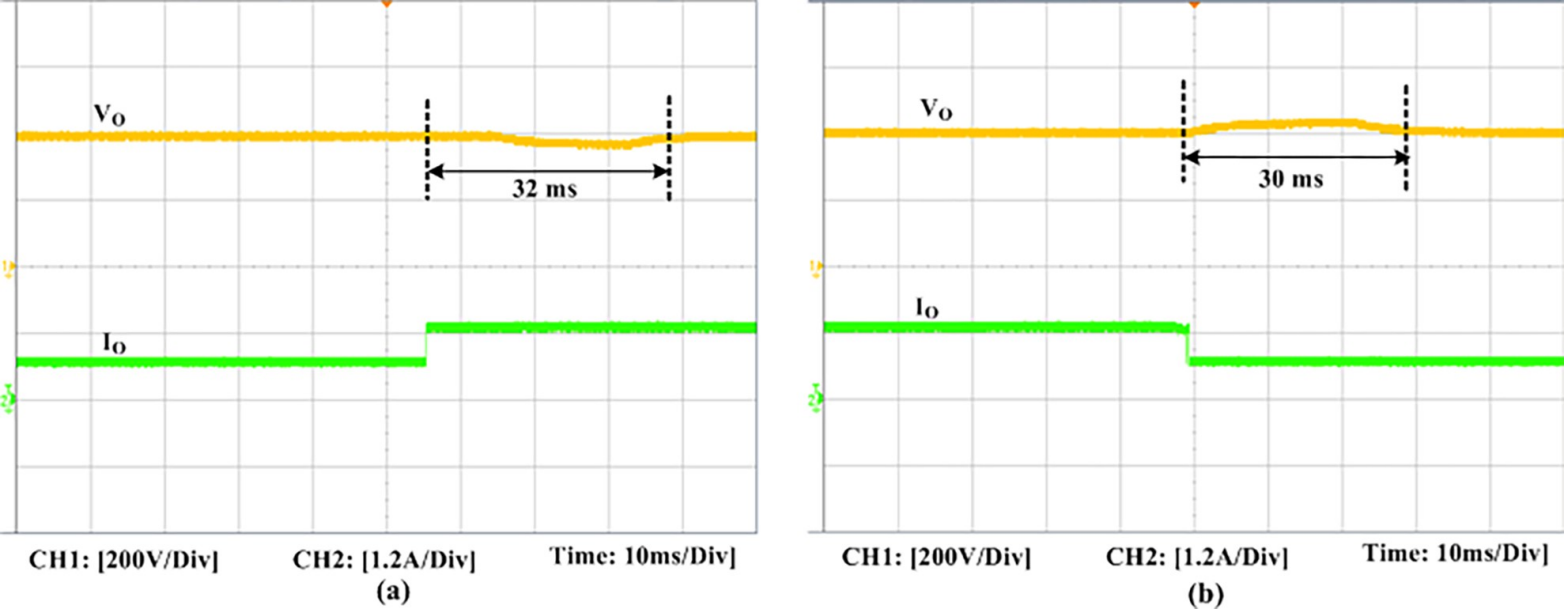
Experimental dynamic response of the PID controller due to load changes. (a) Load increased from 50% to 100%. (b) Load decreased from 100% to 50%.

**Fig 27:**
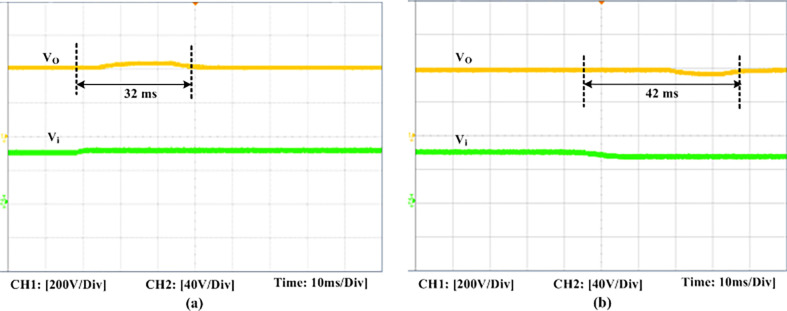
Experimental dynamic responses of the PID controller due to input voltage variations. (a) Input voltage increased from 60V to 70V. (b) Input voltage decreased from 60V to 50V.

**Fig 28:**
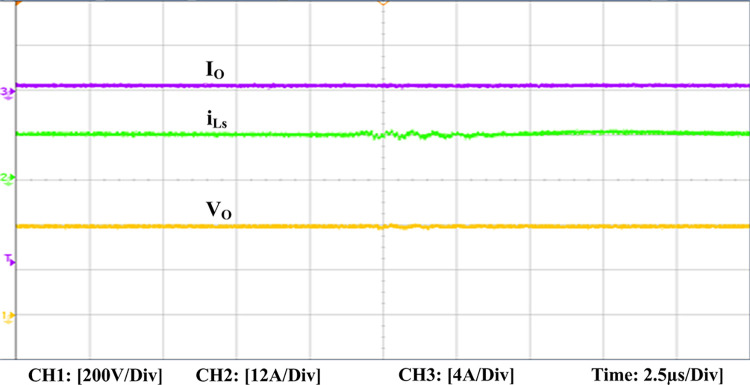
Experimental output voltage, input boost inductor current, and output current.

The experimental findings are displayed in Figs 16–28. Fig 16 represents the gate signals of the four FB MOSFET devices, while Fig 17 displays the *v*_*b*_ and *i*_*r*_. The converter switch operates on ZVS even in the non-resonant condition (*f*_*SL*_ ≠ *f*_*r*_, where *f*_*r*_ is the resonance frequency), and a voltage spike forms across the switch, as illustrated in Fig 18, at this moment, the switching frequency (*f*_*SL*_) is slightly increased, from 80 kHz to 88 kHz. However, if the difference of these frequencies is much higher, for instance, *f*_*SL*_ < *f*_*r*_/2 and *f*_*r*_/2< *f*_*SL*_ < *f*_*r*_, all FB switches operate in a hard-switching state. As a result, lower voltage gain resulting reduced output voltage. In addition, voltage spikes appear at the turn-on and turn-off transitions, as presented in Figs 19 and 20. In Fig 21, the frequency ratio versus voltage gain behaviour is presented, where, voltage gain is 12, at the unity frequency ratio, and then it drops with the frequency ratio declines, and rises at a frequency ratio that is somewhat larger than unity, and subsequently falls again. On the other hand, converter switches operate complete ZVS at the resonant frequency. Figs 22 and 23 depict the ZVS operations of the high-frequency switch *S*_*L1*_, whereas Fig 22 illustrates the ZVS operation during turn-on and turn-off at a 25% load condition. On the other hand, Fig 23 presents the ZVS operation during turn-on and turn-off at 100% load. Similar to this, the other complementary switch, *S*_*L2*_, also operates in the same load range and under ZVS conditions without any incident. Furthermore, the ZVS operations of the low frequency switch *S*_*U1*_ at 25% and 100% load are exposed in Figs 24 and 25, respectively. Additionally, there isn’t any voltage spike in the transitions of the high and low-frequency switches as well.

The dynamic response of this converter for the PID controller to variation in load is represented in Fig 26. When the load is increased from 50% to 100%, the output voltage first slightly drops before increasing for a few milliseconds (ms) to stabilize at the ideal 400 V. It takes about 32 ms (approx.) for the disturbance to stabilize in total. Similar to this, when the load is reduced from 100% to 50%, the output voltage first increases somewhat before stabilizing. Fig 27 displays the PID controller’s dynamic behaviour in the variations of the input voltage. It shows that when the input voltage is slightly increased, the output voltage rises at first before falling back to its starting value and becoming steady. Furthermore, the experimental output voltage (*V*_*o*_), current (*I*_*o*_), and input inductor current (*i*_*Ls*_) are presented in Fig 28. There is good agreement between the experimental and the simulation outcomes.

### 5.2 Loss and efficiency analysis

This subsection provides a concise, step-by-step description of the power loss calculation among the main components and a brief efficiency analysis of the suggested converter.

Initially, the power loss of a MOSFET switching device can be written as follows

$$P_{L(mos)}=P_{s(mos)}+P_{con(mos)}$$ (46)

where *P*_*s(mos)*_ and *P*_*con(mos)*_ are the MOSFET switching and conduction losses, respectively. Since each FB switch of this converter operates ZVS, the switching loss is optimally negligible. Therefore, we can consider only the conduction loss, and it can be stated as:

$$P_{con(mos)}=I_{rms(mos).}^{2}R_{ds(ON)}$$ (47)

The variables $I_{rms(mos)}^{2}$ and *R*_*ds(ON)*_ represents the RMS current flowing through the MOSFET and the ON state resistance of the MOSFET, respectively. Hence, based on the experiential data and datasheet of the C3M0120090D MOSFET utilized in this study, the total power losses of four FB switches can be determined as follows:

$$P_{L(mos)}=4\times (0.92+0.77)=5.63\;W$$ (48)

The power dissipated by a diode can be calculated by multiplying its forward voltage drop, *V*_*F*_, by the average current, *I*_*d(avg)*_, that flows through it during one switching cycle. Therefore, based on the datasheet of the IDH10S120 diode and the average current obtained from the experiment, the diode’s (four diodes) total losses may be determined.


$$P_{L(diode)}={4\times V}_{F}.I_{d(avg)}=4.22\;W$$ (49)

The power dissipation of the B32776G4506K000 film capacitor utilized in this study can be computed using the following formula:

$$P_{\left(cap\right)}=I_{rms(cap).}^{2}{ESR}_{\left(cap\right)}$$ (50)

Hence, the total capacitor power loss will be the sum of four cascaded multiplier capacitors loss and one resonant branch capacitor loss:

$$P_{L(cap)}=4\times P_{\left(cas\_cap\right)}+P_{\left(res\_cap\right)}=3.70\;W$$ (51)

The inductor loss is the sum of the core loss, *P*_*L(core)*_, and the winding loss, *P*_*L(wind)*_. The calculation of *P*_*L(core)*_ involves multiplying the effective volume of the core, *V*_*e*_, by the core loss per unit volume, *P*_*(c/v)*_, according to the following formula:

$$P_{L(core)}={V_{e}.P}_{\left(c/v\right)}$$ (52)

Again, the inductor winding loss can be expressed as:

$$P_{L(wind)}=I_{L(avg)}^{2}.R_{dc}+I_{L(ac-rms)}^{2}.R_{dc}=I_{L(avg)}^{2}.R_{dc}+\frac{I_{L(pk-pk)}^{2}}{value\;of\;AWG}.R_{dc}$$ (53)

The variables *I*_*L(avg)*_, *I*_*L(ac-rms)*_, *I*_*L(pk-pk)*,_ and *R*_*dc*_ represent the average current, ac rms current, peak-peak ripple current magnitude, and winding dc resistance of the inductor, respectively. The VISHAY IHV15BZ500 and two BOURNS JW MILLER 1130-101K-RC devices are utilized in this study as the input boost inductor and resonant branch inductors, respectively. Therefore, based on the testing results and the inductor data sheet, the total inductor losses can be determined as follows:

$$P_{L(ind)}=P_{L(core)}+P_{L(wind)}=4.10\;W$$ (54)

Therefore, the total calculated power loss of the developed converter is

$$P_{LOSS}=P_{L(mos)}+P_{L(diode)}+P_{L(cap)}+P_{L(ind)}=17.60\;W$$ (55)

**Fig 29:**
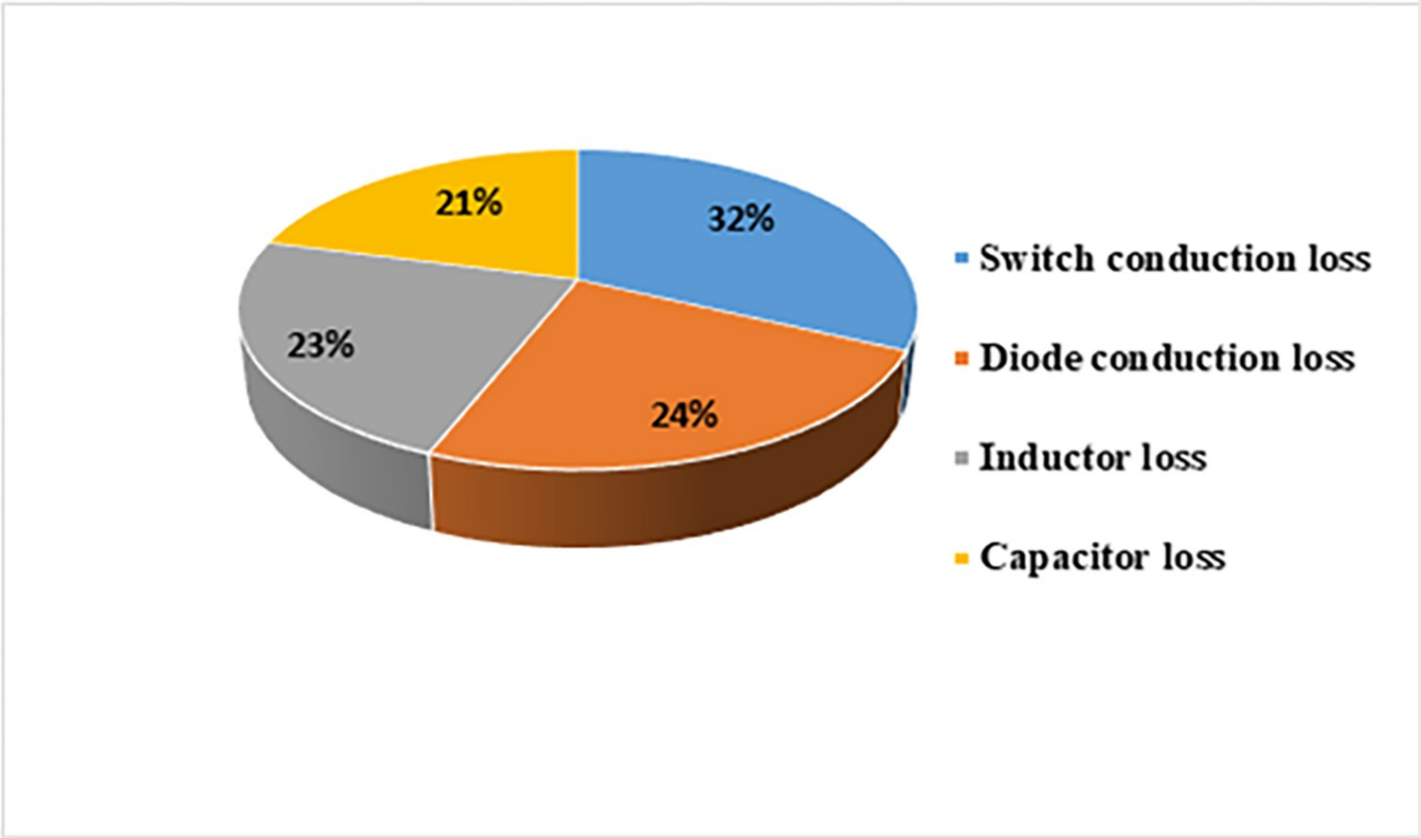
Estimated power losses of different main components used in this converter.

**Fig 30:**
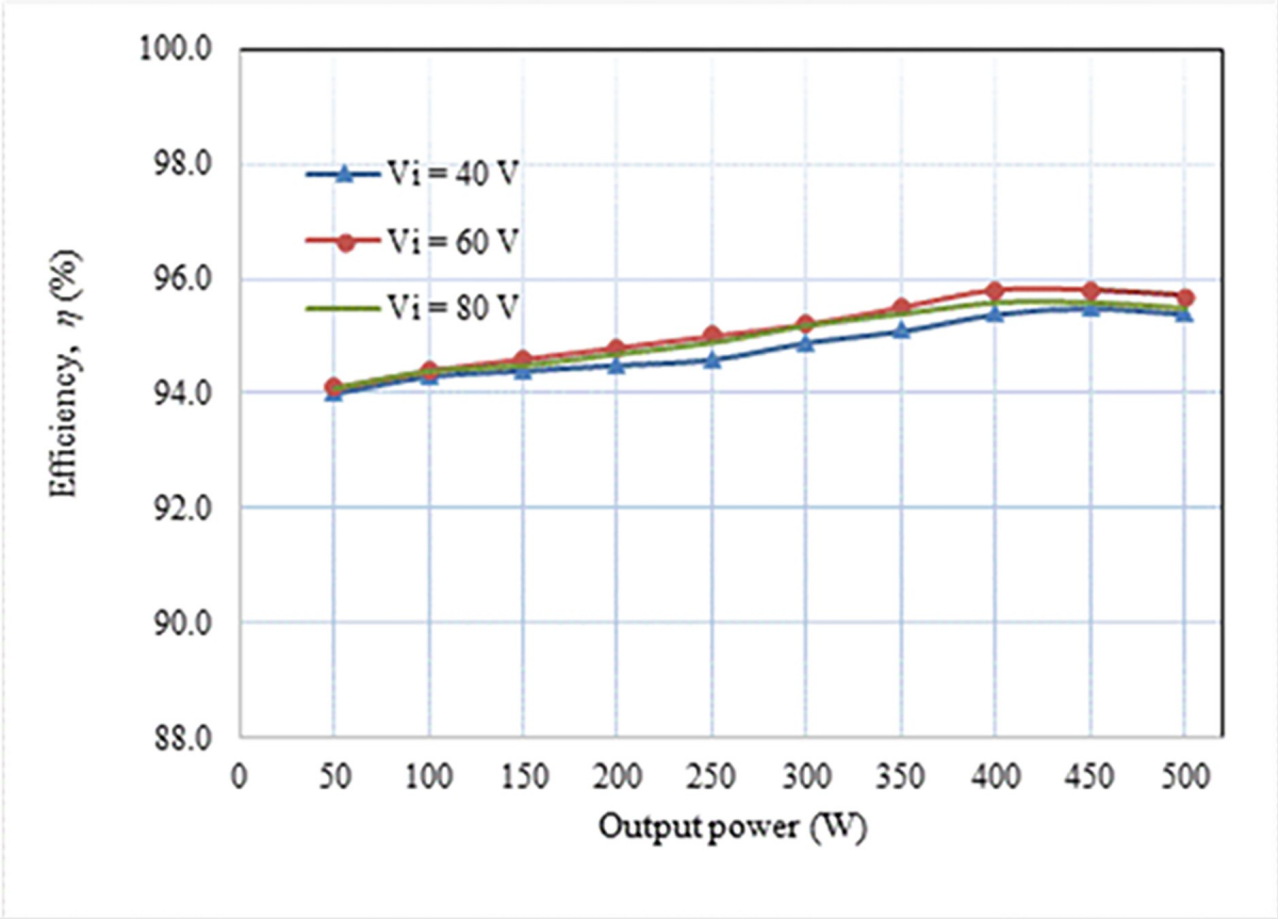
Converter measured efficiency at various loads.

**Fig 31:**
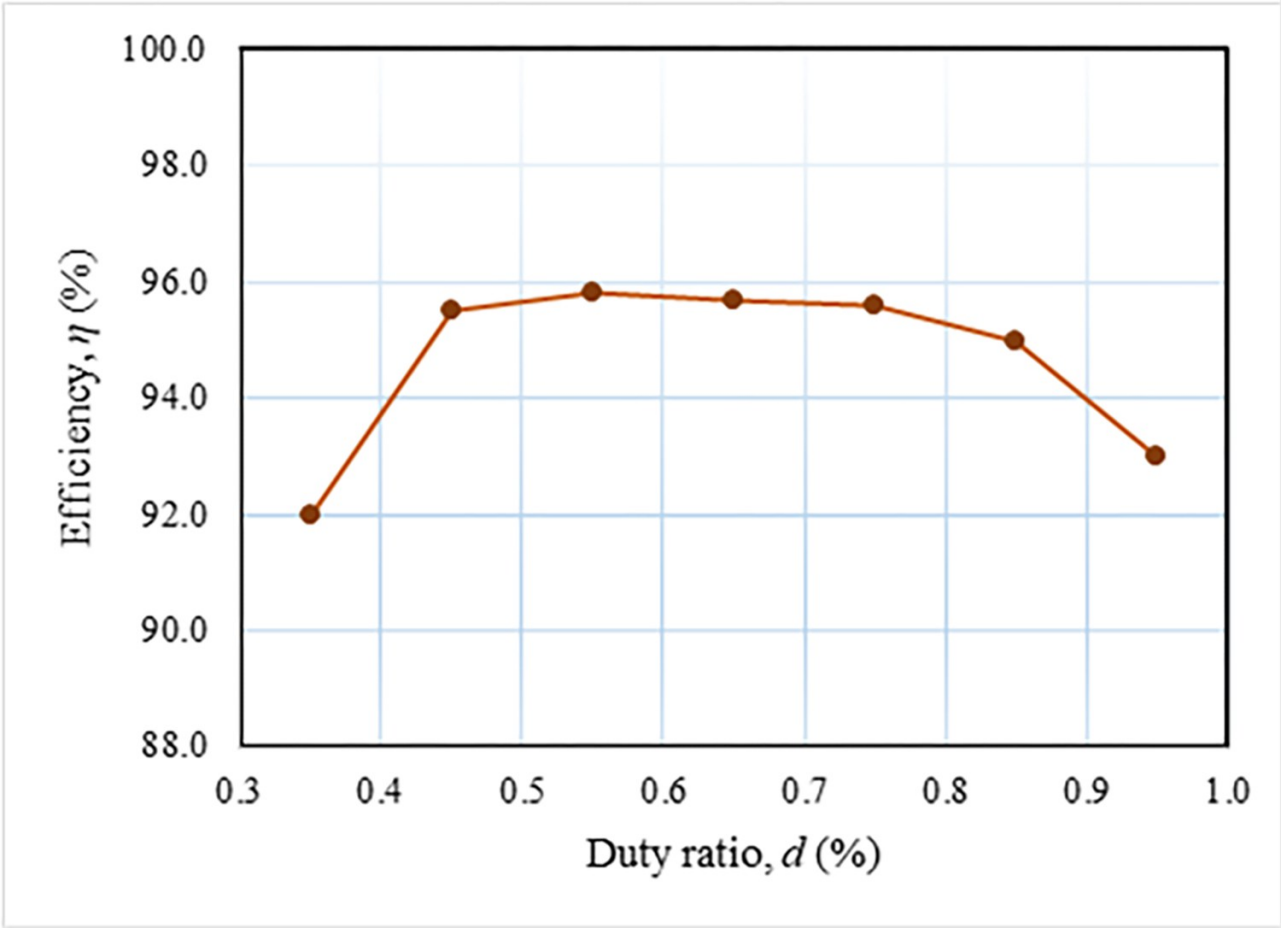
Measured efficiency vs duty cycle.

The experiment yielded a measured power loss of 16.80 W, which is marginally lower than the above-calculated power loss. The power loss for the diode and capacitor is determined based on a temperature of 25°C. Nevertheless, the temperature at the junction of these devices rises as power is dissipated, reducing the diode’s forward voltage drop and the capacitor’s equivalent series resistance (ESR). Consequently, there is a decrease in power loss. The loss distribution of the key apparatuses of the suggested converter is displayed in Fig 29. The switching loss is optimally negligible because every FB switch operates in a ZVS state. On the other hand, the switch conduction loss, which is 32%, is the highest contribution. The inductor and diode losses are close, 23% and 24%, respectively. The least contribution comes from the capacitor, which is 21%. Fig 30 demonstrates the efficiency of the FBRC converter, considering different load conditions and input voltages (40, 60, and 80 V). The input and output currents and voltages are measured using two oscilloscope current probes and multimeters to determine the converter’s efficiency. The system’s peak efficiency is 95.8% when a load of 400 W is connected, and an input voltage of 60 V is applied. This efficiency is achieved when the output voltage is set to 400 V. In addition, Fig 31 presents the effect of the duty ratio on the converter’s efficiency. The converter’s efficiency is also lower at a low duty ratio because the lower output voltage causes higher current resulting in more power loss. Again, at a higher duty ratio, converter efficiency is also lower.

## 6. Conclusion

High-gain dc-dc converters play a significant role in various renewable energy and other systems. This study introduces a novel ZVS full-bridge cascaded step-up dc-dc converter. In addition to comprehensive analytical analysis, a laboratory-scale prototype was designed, constructed, and subjected to experimental testing. The theoretical findings closely align with the observed experimental outcomes of the 500 W prototype converter.

In the prototype implementation, high-performance SiC-based MOSFETs have been utilized as switching devices. Furthermore, the PID-based PWM control scheme and the resonant component have been employed to control the turn-off and turn-on operations of the FB switches from 25 to 100% load conditions facilitating zero-voltage switching (ZVS). The application of four such FB switches ensured complete soft-switching (zero switching loss). Additionally, the resonant technique leads to smaller passive components and reduces voltage stress on both active and passive devices. Consequently, this results in smaller and more cost-effective devices compared to other converters. Moreover, it offers less ripple in input current (5%) and output voltage (0.76%). From an efficiency standpoint, the converter achieves its maximum efficiency of 95.8% when operated at input and output voltages of 60 V and 400 V, respectively, with a load power of 400 W.

The dc-dc converter proposed in this research achieves an excellent dc voltage conversion ratio (> 10) by preventing excessively high-duty cycle operation of the MOSFET switches, thereby limiting the maximum current flow through the active devices. Hence, the proposed FBRC converter is well-suited for systems generating low output voltage, making it particularly suitable for variable wide input ranges (tested from 40 V to 80 V), commonly found in low voltage systems like photovoltaic (PV), fuel cell (FC), and electric vehicles (EV).
